# Light-Controlled Folding and Chiroptical Sensing of G‑Quadruplex DNA

**DOI:** 10.1021/acs.jpclett.6c01816

**Published:** 2026-07-31

**Authors:** Aleksandra Matusiak, Jakub Trojnar, Katarzyna Matczyszyn, Marco Deiana, Marta Dudek

**Affiliations:** Institute of Advanced Materials, 49567 Faculty of Chemistry Wrocław University of Science and Technology, Wyb. Wyspiańskiego 27, 50-370 Wrocław, Poland

## Abstract

Combining reversible optical control with structural sensing of G-quadruplex (G4) DNA remains a significant challenge for nucleic acid-targeting photoswitches. Herein, we report the synthesis and photophysical characterization of a visible-light-responsive *ortho*-fluoroazobenzene derivative, **exAzoPy**_**2**_, designed to modulate G4 DNA structures. The interaction of **exAzoPy**_**2**_ with unfolded and folded G4 topologies was investigated using circular dichroism (CD) and fluorescence spectroscopy. The photoswitch exhibits isomer-dependent effects on antiparallel G4 structures: the *cis*-rich photostationary state promotes G4 folding, whereas photoinduced *cis*-to-*trans* isomerization favors the unfolded state. In addition to structural modulation, **exAzoPy**_**2**_ generates topology- and isomer-dependent induced circular dichroism (ICD) signals in the ligand absorption region. Distinct ICD responses depend on DNA conformation and G4 topology, enabling chiroptical discrimination of different G4 states. The combination of visible-light responsiveness, reversible structural modulation, and ICD-based spectroscopic readout highlights **exAzoPy**_**2**_ as a photoswitch capable of both controlling and sensing different G4 DNA architectures.

## Graphical abstract

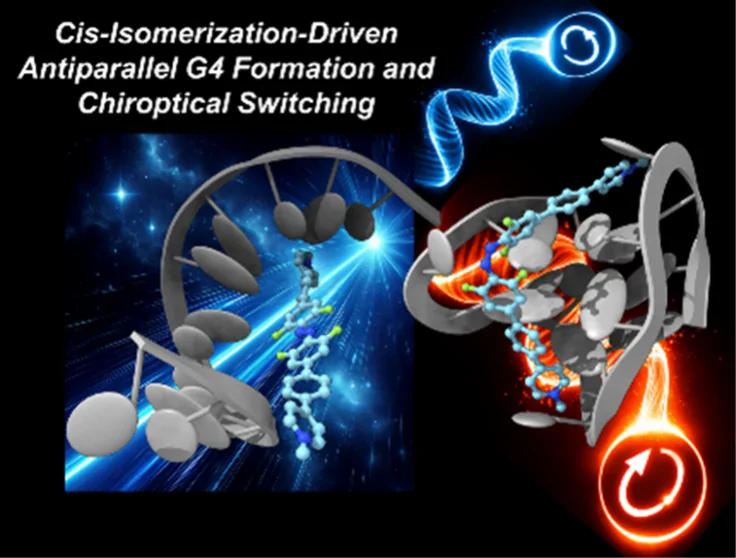

The widespread availability and precise controllability of light make it an attractive stimulus for driving energy transduction, advanced materials fabrication, and responsive molecular systems.^1–3^ Compared with chemical stimuli, light can be delivered with high spatial and temporal precision, does not introduce additional chemical species into the system, and can be finely controlled through wavelength selection.^4,5^ Taken together, these features make light an exceptionally powerful external trigger for controlling molecular processes.^6^ Organic chemistry provides a broad range of synthetic molecular systems that can be reversibly controlled by light, commonly known as molecular photoswitches.^7^ Upon irradiation at defined wavelengths, these compounds undergo reversible isomerization, enabling remote and dynamic control over coupled systems.^3,8,9^ Owing to this ability, photoresponsive molecules have attracted considerable attention across chemistry, biology, and materials science.^10,11^ Their applications span molecular data storage and sensing, as well as the light-mediated control of biomolecular structure and function.^12^ Among biomolecular targets, oligonucleotides are particularly appealing targets.^6^ Photoswitches can either be covalently incorporated into DNA strands or interact noncovalently with nucleic acids to modulate their structure.^6^

In this context, G-quadruplexes (G4s) have received special attention.^3,10,13,14^ These structures are formed by the stacking of guanine tetrads (G-quartets), stabilized by Hoogsteen hydrogen bonding and monovalent cations such as K^+^ or Na^+^.^14–16^ G4s are characterized by high thermodynamic stability, well-defined structures, and remarkable topological polymorphism, including parallel, antiparallel, and hybrid arrangements.^17^ Interest in G4 structures has increased markedly due to their proposed involvement in key biological processes, particularly gene regulation, telomere maintenance, and pathways associated with aging and disease.^14,18,19^ Beyond their biological significance, G4s possess an intrinsic ability to form higher-order assemblies, rendering them attractive structural motifs for DNA-based nanotechnology and functional nanodevices.^20,21^ Given both their structural polymorphism and biological relevance, the development of small photochromic molecules capable of targeting G4 DNA has emerged as a powerful strategy to modulate their stability, folding, and topology.^3,10^ Among the various photoswitches, azobenzene (Azo) stands out due to its photochemical robustness, synthetic accessibility, and structural tunability. Its photophysical properties, including absorption characteristics and the thermal half-life of the *cis* isomer, can be readily adjusted through chemical modification, making Azo-based systems highly adaptable for applications in light-controlled biomolecular regulation.^8,10,22,23^

The dynamic behavior of G4 structures, including their bending and stretching motions, has been explored for nearly three decades.^24^ A pioneering strategy for light-controlled manipulation of G4 architecture was introduced by Ogasawara and Maeda, who covalently incorporated Azo derivatives into nucleic acids to regulate conformational changes via photoisomerization.^25^ This seminal work demonstrated the feasibility of modulating DNA structure using photochromic molecules. Subsequently, Zhou and co-workers reported the first noncovalent strategy for light-controlled modulation of G4 structures.^26^ Distinct isomeric states of Azo ligands displayed different binding modes and selectively promoted folding or unfolding of parallel G4s, establishing a versatile platform for reversible and remote regulation without permanent modification of the oligonucleotide.^26^ Later studies further demonstrated light-driven interconversion between distinct G4 conformations,^27^ findings expanded by Galan and co-workers.^28,29^ In parallel, Azo-G4 complexation was shown to generate induced circular dichroism (ICD) signals within the ligand absorption region, whose intensity depends on the isomeric state of the photoswitch.^30,31^ *Ortho*-fluoroAzo derivatives, in particular, were found to exhibit pronounced isomer-dependent effects on G4 stabilization^32^ and even enable photoregulation of G4 structures in live cells.^33^ For comprehensive discussions of reversible photoregulation of DNA structures, including G4s, the reader is referred to recent review articles.^3,6,34^ Despite this significant progress, important challenges remain. Photoactive small-molecule systems capable of delivering (i) topology-selective modulation, (ii) efficient visible-light responsiveness with favorable photostationary states, (iii) reversible switching, and (iv) spectroscopic readout of structural discrimination remain limited.

Motivated by these considerations, we sought to develop a visible-light-responsive photoswitch capable of topology-sensitive modulation of G4 DNA. To achieve this, we designed **exAzoPy**_**2**_, an *ortho*-fluoroAzo derivative functionalized in the *para* positions with 1-methyl-4-phenylpyridinium groups selected to promote electrostatic and π–π interactions with G4 DNA. The molecular design was based on the premise that isomer-dependent differences in geometry and electronic structure would translate into distinct binding modes and differential effects on G4 folding. The photophysical properties of **exAzoPy**_**2**_ were characterized by UV–Vis spectroscopy and NMR analysis. Its interactions with parallel and antiparallel G4 topologies, as well as with their corresponding unfolded states, were investigated using circular dichroism (CD), fluorescence titrations, and thermal denaturation experiments. Our photoswitch exhibits pronounced isomer-dependent behavior, with the *cis*-rich photostationary state (PSS) promoting folding of antiparallel G4 structures, while the *trans* isomer favors their unfolding state. Beyond structural modulation, the system generates a topology- and isomer-dependent ICD signal within the ligand absorption region, enabling direct chiroptical discrimination between distinct DNA conformations. The combination of reversible light-driven structural control and simultaneous spectroscopic readout within a single small-molecule framework provides a powerful platform for mechanistic investigations of G4 dynamics. More broadly, this strategy provides a foundation for further studies toward light-controlled regulation of nucleic acid structures and the development of photoresponsive probes for topology-sensitive G4 investigations.

The ability to be operated with light that features in different properties of isomers makes Azo a tempting scaffold that can be used to influence DNA structure and functions.^3^ Therefore, we aimed to design a photoswitchable molecule, named **exAzoPy**_**2**_, that can be fully controlled with visible light (Scheme 1 and Figure 1A). It has been shown in the literature that the modification of the *ortho* positions of the Azo core results in n−π* band separation for the two isomers, allowing manipulation by visible light.^35^ Due to the advantageous properties resulted by fluorine substitutions^36^ and our strong experience in working with this type of compound,^8,31,32^ we decided to obtain *ortho*-fluoroAzo derivatives with an extended aromatic system, potentially enhancing π-stacking interactions with the G-tetrad platform.^37^ Moreover, we choose the pyridinium group as it is known for its strong interaction with the G4 template.^23,38^

**Scheme 1:**
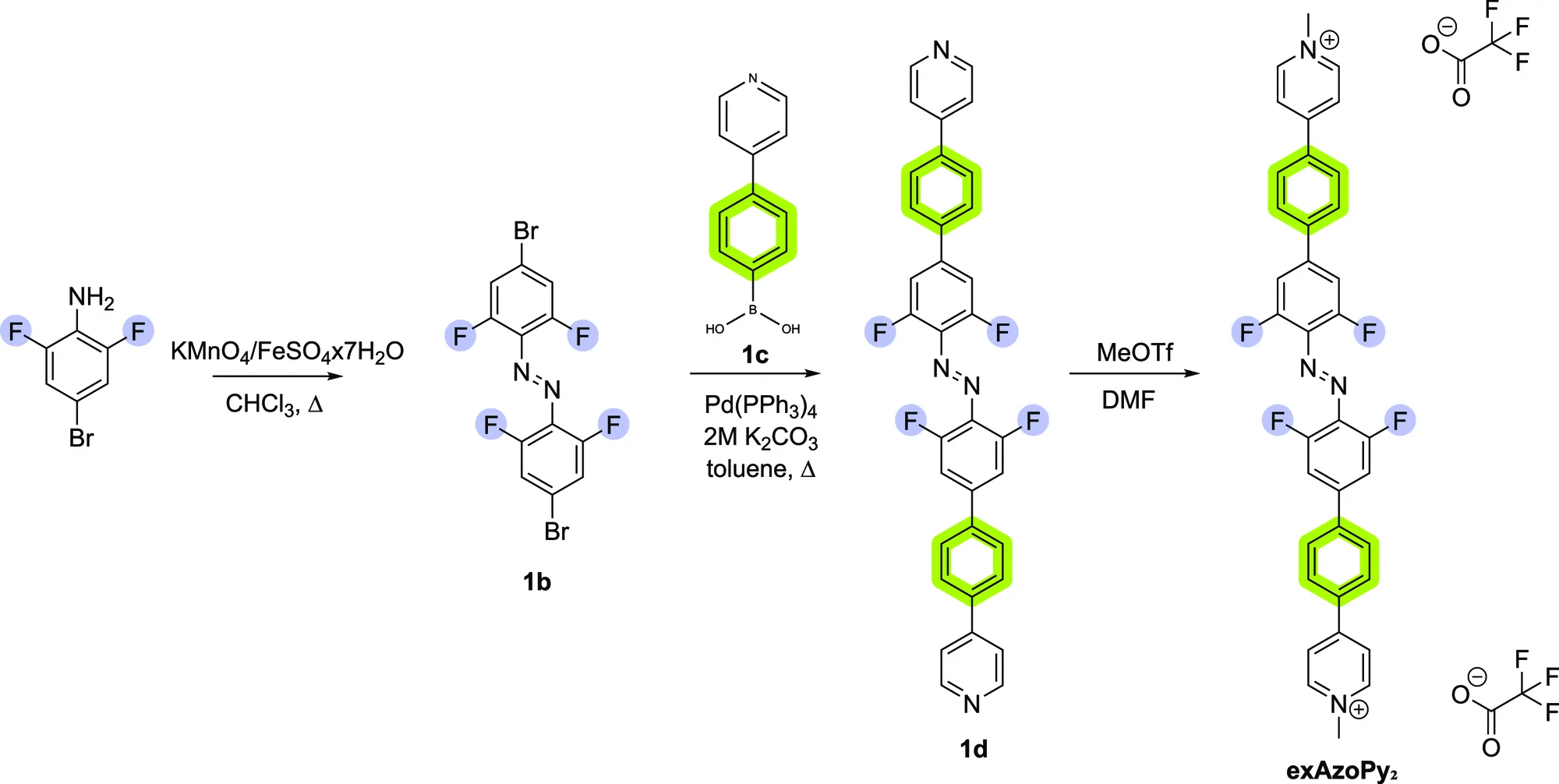
Synthetic Route of **exAzoPy**_**2**_

**Figure 1:**
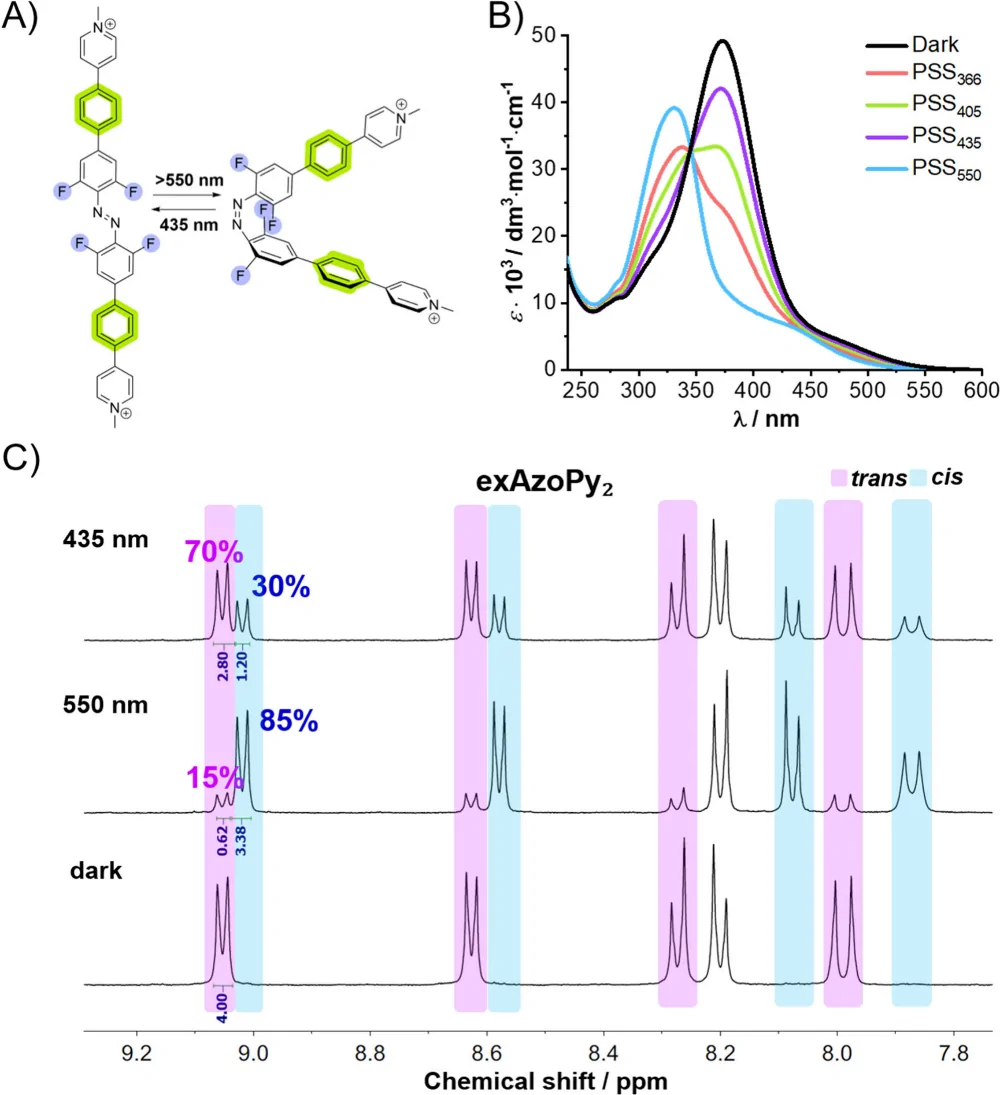
(A) Chemical structures of **exAzoPy**_**2**_ in *trans* or *cis* forms. (B) Absorption spectra of **exAzoPy**_**2**_ recorded in 10 mM lithium cacodylate buffer after irradiation with light of different wavelengths (c_**exAzoPy** **2**_ = 20 μM). (C) Quantification of the PSSs of **exAzoPy**_**2**_ (c_**exAzoPy2**_ = 3.2 mM, in DMSO-*d*_6_ at 25 °C) by ^1^H NMR spectroscopy. The contents of *trans* and *cis* isomers in solution were calculated from the intensity ratios of the integrals of the corresponding signals.

The visible light–responsive compound **exAzoPy**_**2**_ was synthesized through a three-step synthetic route (Scheme 1). In the first step, a well-established homo-oxidative coupling protocol was employed using 4-bromo-2,6-difluoroaniline as the starting material.^39^ This transformation afforded the corresponding Azo intermediate **1b** in 22% yield. Subsequently, the obtained Azo derivative **1b** was subjected to a Suzuki cross-coupling reaction with compound **1c**, leading to the formation of the compound **1d**, which was used in the next step without purification.^23^ In the final step, quaternization was achieved via methylation using methyl trifluoromethanesulfonate (MeOTf) in dimethylformamide (DMF). The reaction proceeded cleanly, and the target compound **exAzoPy**_**2**_ was isolated after purification by reversed-phase chromatography using a water/acetonitrile mixture containing trifluoroacetic acid (TFA) as an additive. The product was obtained as the TFA salt in 25% yield over two steps and fully characterized by NMR spectroscopy, HRMS, and HPLC analysis (SI pp. S5–S8).

With **exAzoPy**_**2**_ in hand, we initiated our investigations by examining its photophysical properties (Figure 1). The UV/vis absorption spectrum of **exAzoPy**_**2**_ in its thermodynamically stable *trans* form displays the characteristic features of Azo derivatives, including a pronounced absorption band centered at 360 nm, which can be assigned to the π–π* transition.^40^ Upon irradiation with green light (550 nm), efficient *trans*→*cis* photoisomerization is induced, as evidenced by distinct changes in the absorption spectrum. At the PSS, an intense band with a maximum at 330 nm, attributed to the π–π* transition of the *cis* isomer, is observed alongside a weaker band at 430 nm corresponding to the n−π* transition (Figures 1B and S5). Although *ortho*-fluoroAzo derivatives are typically characterized by a clear separation of the n−π* bands of the two isomers, enabling efficient bidirectional switching using exclusively visible light,^35^ such a clear separation is not apparent for **exAzoPy**_**2**_. This behavior can likely arise from a bathochromic shift of the *trans* isomer absorption, which can be attributed to the extended π-conjugated aromatic framework of the molecule. Nevertheless, ^1^H NMR spectroscopy confirmed efficient photoswitching and a favorable isomer distribution at the respective PSSs (Figure 1C). Irradiation with green light (550 nm) afforded 85% of the *cis* isomer, whereas subsequent irradiation with blue light (435 nm) regenerated 70% of the *trans* isomer (Figure 1C). Consistent with literature reports on *ortho*fluoroAzo,^36^ the *cis* isomer exhibits high thermal stability at ambient temperature, a feature particularly advantageous for applications requiring light-controlled switching without rapid thermal relaxation. Indeed, Arrhenius analysis revealed a thermal half-life of approximately 236 h at room temperature for the *cis* isomer of **exAzoPy**_**2**_ (See SI pp. S9).

Inspired by the seminal work of Zhou and co-workers on the photoregulation of G4 DNA,^26^ we next investigated whether **exAzoPy**_**2**_ can act as a light-responsive effector capable of modulating oligonucleotide folding in a topology-selective manner (Figures 2 and S7). CD spectroscopy was employed as a primary analytical tool, given its sensitivity to G4 topology and its ability to distinguish between parallel, antiparallel, and hybrid conformations.^41^ We examined a representative set of DNA sequences (Table S2) known to adopt parallel (*c-MYC* Pu22, *c-MYC* Pu24T, *VEGF*), and antiparallel (TBA, Bom17) G4 topologies in the presence of K^+^ ions. In their unfolded state (no K^+^ ions), all sequences displayed CD spectra of low intensity, consistent with the absence of ordered secondary structure (Figures 2A,B and S7). Addition of the *trans* isomer of **exAzoPy**_**2**_ did not induce the characteristic CD signatures of G4 folding for any of the investigated sequences (Figure S7A,C,E,G,I). This observation suggests that the elongated, planar geometry of the *trans* isomer does not favor productive interactions with the oligonucleotides under these conditions. A similar absence of structural induction was observed for sequences predisposed to form parallel G4 topologies when treated with the *cis*-rich PSS of **exAzoPy**_**2**_ (Figure S7B,D,F). This indicates that neither isomer was able to effectively induce folding into the parallel G4 topology. In striking contrast, a pronounced and reproducible folding response was observed for sequences capable of adopting antiparallel G4 conformations. Upon addition of the *cis*-rich PSS of **exAzoPy**_**2**_ to unfolded TBA and Bom17 oligonucleotides (Figure 2A,B), the CD spectra evolved to display features characteristic of antiparallel G4 structures. Specifically, a positive band around 290 nm and a negative band near 270 nm emerged, closely resembling the spectral signature observed under 100 mM K^+^ conditions (Figure 2A,B). Importantly, these structural signatures were absent in the presence of the *trans* isomer (Figure S7G,I), underscoring the isomer-specific nature of the interaction. The distinct behavior of *cis* isomer can be rationalized by its bent geometry and reduced end-to-end distance, which may facilitate multivalent interactions with antiparallel G4 structures, as antiparallel topology is characterized by distinct loop arrangements and groove dimensions compared to parallel G4s.^17^ In contrast to earlier systems,^26^ where the *trans* isomer promoted telomeric G4 formation, our photoswitch exhibits complementary behavior: the *cis* state selectively promotes antiparallel G4 formation, highlighting how subtle differences in molecular design translate into distinct topological outcomes.

**Figure 2:**
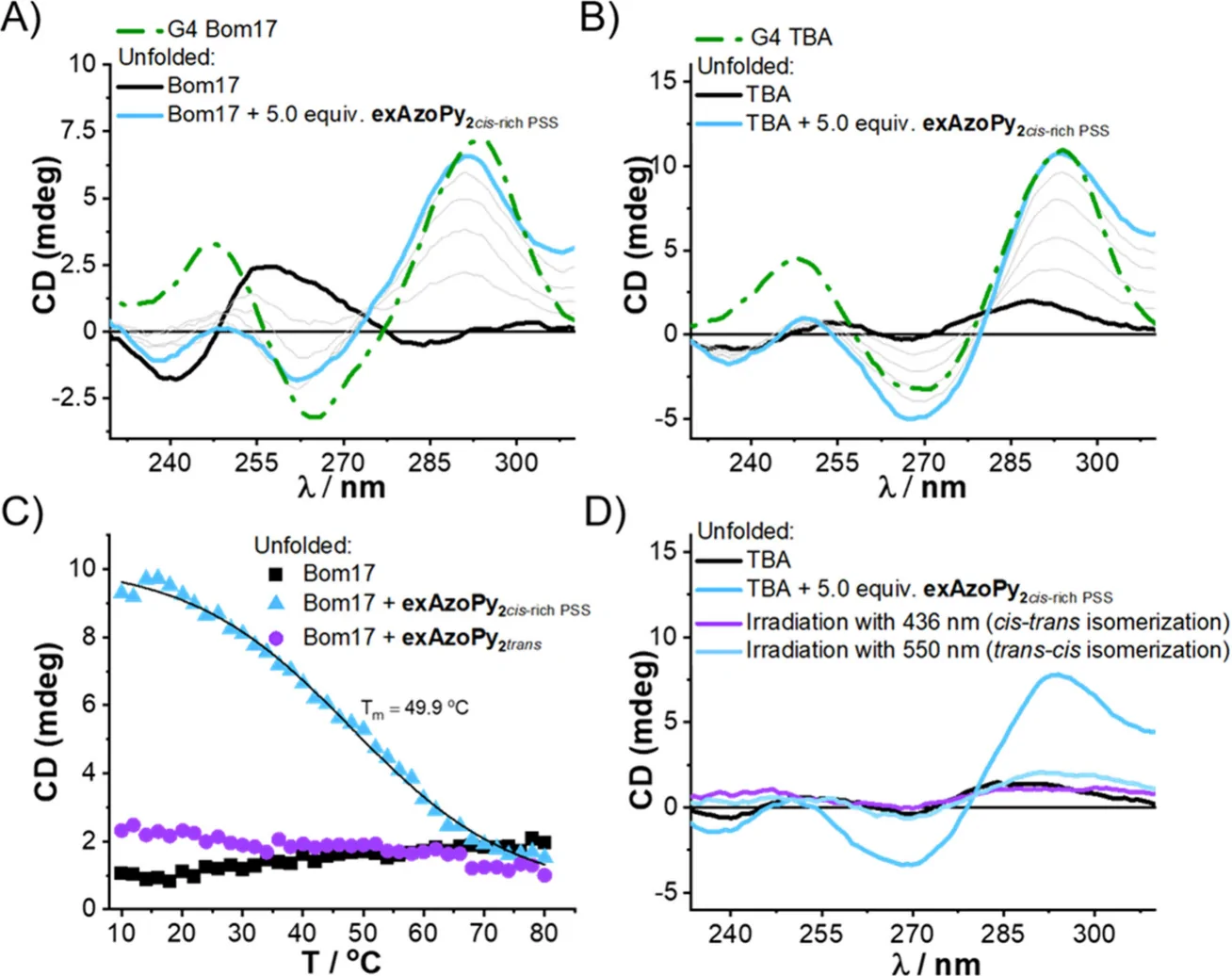
CD titration of the unfolded Bom17 (A) or TBA (B) with **exAzoPy**_**2** *cis*‑rich PSS_. Experimental conditions: c_DNA_ = 2 μM, c_cacodylate_ = 10 mM, c_photoswitch_ = from 0 μM to 10 μM. (C) Melting profiles of unfolded Bom17 alone and in the presence of *trans*/*cis*-rich PSS of **exAzoPy**_**2**_. Experimental conditions: c_DNA_ = 2 μM, c_cacodylate_ = 10 mM, c_photoswitch_ = 10 μM. The CD signal was plotted as a function of temperature at 292 nm. Results are presented as the average of two independent experiments. (D) Photoisomerization of the unfolded TBA in the presence of the **exAzoPy**_**2**_.

It should be noted that the ligand-induced folding experiments were performed under salt-free conditions, which are not intended to reproduce the intracellular ionic environment, where K^+^ strongly promotes G4 stabilization. Instead, these conditions provide a deliberately stringent model for evaluating whether the *cis* isomer can actively induce folding of an otherwise unstructured G-rich sequence. This distinction is important when considering potential biological applications. In cells, G4-forming sequences are highly dynamic rather than static structures, and their folding state is influenced by DNA replication and transcription, chromatin accessibility, R-loop formation, transient exposure of single-stranded DNA, and the activity of G4-binding and G4-resolving proteins. Therefore, the biological relevance of the present system is best considered in the context of transiently exposed, weakly folded, or protein-remodeled G-rich sequences, rather than constitutively folded G4 structures under fully stabilizing ionic conditions.

To substantiate the influence of the photoswitch on unfolded oligonucleotides, which tends to form antiparallel G4 structures in the presence of the salt, we performed CD melting experiments (Figures 2C and S8). Temperature-dependent CD measurements were recorded for the unfolded state of TBA and Bom17 in the absence or presence of the *trans* or *cis*-rich PSS of **exAzoPy**_**2**_. Upon addition of the *cis*-rich PSS of **exAzoPy**_**2**_ to the unfolded oligonucleotide, well-defined CD signatures characteristic of antiparallel G4 secondary structures emerged, and as expected, these two sequences displayed cooperative melting transitions with *T*_m_ values of 49.9 °C for Bom17 and 54.3 °C for TBA (Figures 2C and S8). These values are in good agreement with those observed for G4 sequences folded in the presence of K^+^ ions (TBA, Bom17),^32^ clearly indicating the formation of thermodynamically stable G4 structures. In contrast, the melting profiles obtained in the presence of the *trans* isomer closely resembled those of unfolded oligonucleotides, confirming that this isomer neither induces nor stabilizes antiparallel G4 structures (Figures 2C and S8). Next, the folding and unfolding dynamics of TBA under photochemical control were investigated (Figure 2D). G4 folding was induced in the presence of the *cis*-rich PSS of **exAzoPy**_**2**_. Subsequent irradiation at 435 nm triggered isomerization to the *trans* state and resulted in dissociation of the folded G4 structure. Notably, irradiation at 550 nm did not restore G4 folding from the stretched state, indicating a unidirectional modulation of DNA properties, as previously observed for other Azo derivatives interacting with G4 DNA.^23,32,33^ All these findings support a mechanism in which photoisomerization of **exAzoPy**_**2**_ directly modulates its structural compatibility with antiparallel G4 architectures. Collectively, our results demonstrate that **exAzoPy**_**2**_ functions as a topology-selective, light-responsive effector that preferentially induces and stabilizes antiparallel G4 DNA in its *cis* state. The reversible interconversion between the inactive (*trans*) and active (*cis*) states provides a molecular basis for photoregulated control over DNA secondary structure, offering opportunities for spatiotemporal manipulation of G4 formation using visible light.

Building on the CD titration studies of **exAzoPy**_**2**_ with unfolded oligonucleotides, in which ICD signals were observed within the absorption region of the ligand (Table 1), we next investigated the behavior of the photoswitch in the presence of distinct G4 topologies, namely parallel and antiparallel structures (Tables 1 and S2). This analysis was undertaken to evaluate whether **exAzoPy**_**2**_ can serve as a chiroptical reporter of DNA conformational changes.

**Table 1:** Presence (Yes) or Absence (No) of ICD Bands for Complexes of the *cis*-Rich PSS or *trans* Isomer of **exAzoPy**_**2**_ with Unfolded and Folded G4 DNA

G4 topology	Sequence	Isomer	ICD of G4	ICD of unfolded DNA
antiparallel	TBA	*cis*	yes	yes, but G4 generated
	Bom17	*trans*	no	yes
parallel	*c-MYC* Pu22	*cis*	no	yes
	*c-MYC* Pu24T	*trans*	no	yes
	VEGF			

As a first step, the binding affinities of the *trans* and *cis* isomers of **exAzoPy**_**2**_ toward G4 structures were determined (Table S2) using a fluorescence quenching assay (Figure S9). The study was performed with a Cyanine 5 (Cy5)-labeled TBA and *c-MYC* Pu22, in which fluorescence quenching occurs upon ligand binding in proximity to the 5′-terminal G-tetrad. Both isomers exhibited robust binding to the G4 templates, with association constants of 1.1 × 10^6^ M^–1^ (TBA) and 5.0 × 10^6^ M^–1^ (*c-MYC* Pu22) for the *cis*-rich PSS, and 2.2 × 10^6^ M^–1^ (TBA) and 9.1 × 10^6^ M^–1^ (*c-MYC* Pu22) for the *trans* isomer. The higher affinity of the *trans* isomer can be attributed to its more planar structure, which promotes efficient π–π stacking interactions with the G-tetrads. In contrast, the nonplanar geometry of the *cis* isomer likely reduces the extent of π-stacking, resulting in a binding mode that partially engages the G-tetrad surface and relies more heavily on groove binding and electrostatic interactions.

Having established that the *cis*-rich PSS of the photoswitch promotes folding and stabilization of antiparallel G4 structures (Figures 2 and S8), we next investigated whether a comparable effect could be observed for prefolded antiparallel G4s (Figure S10). To this end, CD-based thermal melting experiments were performed, monitoring the temperature-dependent changes in molar ellipticity for Bom17 and TBA in the absence and presence of the respective photoswitch isomers (Figure S10). As anticipated, thermal denaturation studies revealed a pronounced stabilization of antiparallel G4 structures in the presence of the *cis*-rich PSS (Figure S10). The change in melting temperature (ΔT_m_) for the Bom17-**exAzoPy**_**2**_ and TBA-**exAzoPy**_**2**_ complexes reached at least 29.6 and 23.8 °C, respectively. These values may represent conservative estimates because partial thermal relaxation of the *cis*-rich PSS to the *trans* isomer can occur during the melting experiment. In contrast, addition of the *trans* isomer resulted in only negligible changes in the melting temperature (ΔT_m_ = 1.3 °C for Bom17 and 2.5 °C for TBA). These findings further confirm the isomer-dependent behavior of **exAzoPy**_**2**_ and demonstrate that the *cis*-rich form selectively stabilizes antiparallel G4 conformations compared to the *trans* isomer.

Encouraged by the strong and isomer-dependent binding behavior of **exAzoPy**_**2**_, as well as the ICD signals observed for unfolded oligonucleotides (Table 1, Figures 3A, B and S7), we next explored its potential as a chiroptical reporter of G4 DNA structures. Our investigation focused on both parallel and antiparallel G4 topologies (Tables 1 and S2).

**Figure 3:**
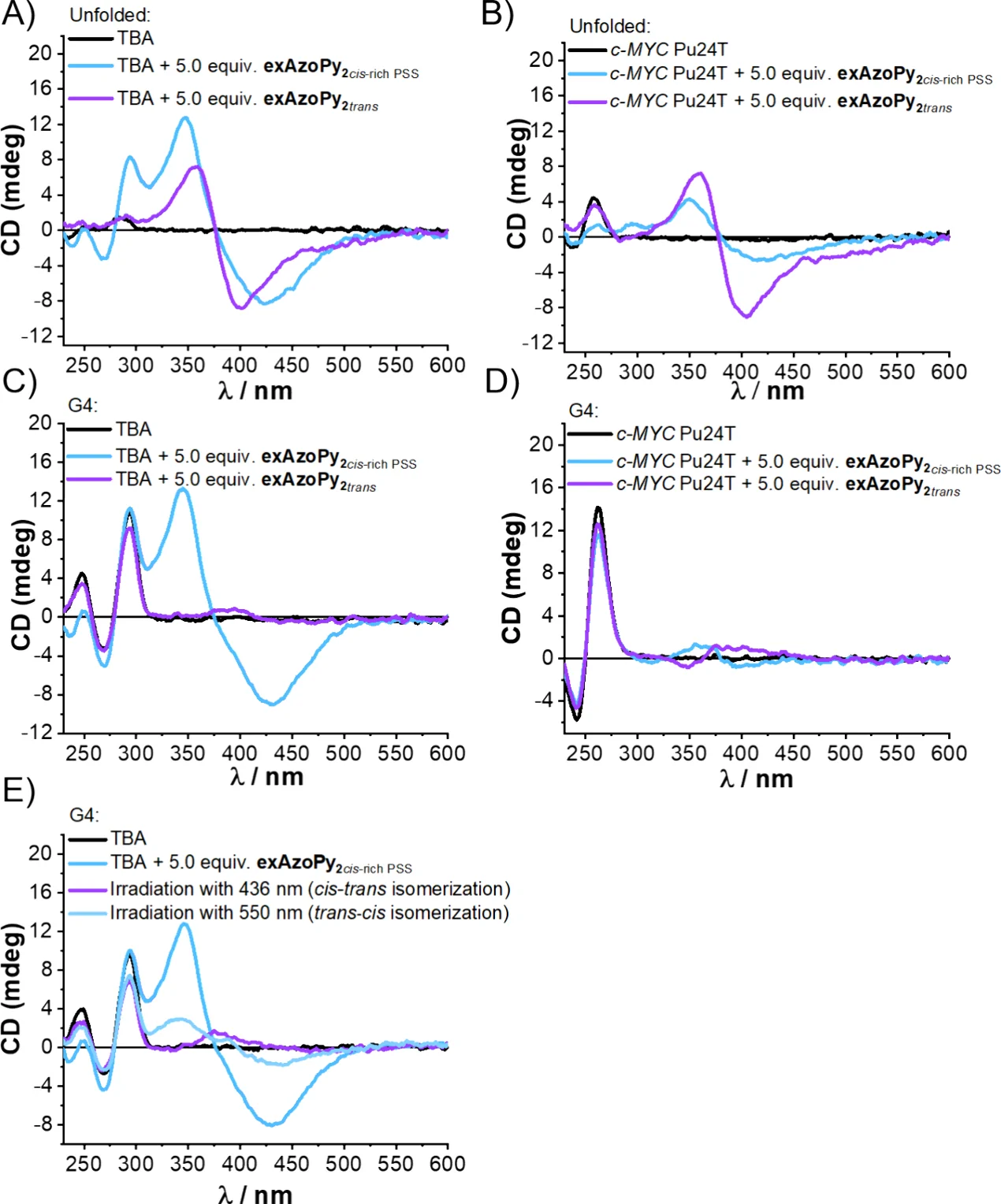
CD spectra of the unfolded TBA (A), *c-MYC* Pu24T (B), and the corresponding G4 of TBA (C) and *c-MYC* Pu24T (D), recorded in the absence and presence of the *trans/cis*-rich PSS of **exAzoPy**_**2**_. Experimental conditions: c_DNA_ = 2 μM, c_tris(unfolded)/cacodylate (G4)_ = 10 mM, c_photoswitch_ = 10 μM, for G4 samples, 100 mM KCl was added. (E) Photoisomerization of the TBA G4 structure in the presence of **exAzoPy**_**2**_ upon sequential irradiation at 435 nm followed by 550 nm.

Specifically, we monitored ICD signals in the ligand absorption region to evaluate whether distinct G4 conformations generate characteristic spectroscopic fingerprints. Under physiological conditions, a pronounced ICD response was observed exclusively for complexes formed between the *cis*-rich PSS of **exAzoPy**_**2**_ and antiparallel G4 structures (Table 1; Figures 3C and S11A). Importantly, addition of the *cis*-rich PSS to unfolded Bom17 and TBA induces their folding into antiparallel G4 structures; thus, in these cases, the emergence of the ICD band reflects the chiral arrangement of the ligand associated with the newly formed G4 architecture, analogous to that observed for prefolded antiparallel G4s (Figures 3A and S7J). In contrast, no ICD signal was detected for complexes of antiparallel G4s with the *trans* isomer under identical conditions (Figures 3C and S11A). Interestingly, the *trans* isomer displayed a distinct discrimination pattern (Table 1). A well-defined ICD band appeared for the *trans*-**exAzoPy**_**2**_ complex with unfolded DNA (Figures 3A,B and S7), demonstrating that the *trans* form differentiates between folded and unfolded states and produces a chiroptical response selectively in the latter. In the case of prefolded parallel G4 topology, neither isomer generated a significant ICD response (Figures 3D and S11B,C). The well-structured, exciton-coupled bisignate ICD bands observed in several cases likely arise from short-range intermolecular coupling between adjacent Azo chromophores.^42^ Notably, such bisignate signals were consistently detected for complexes of the *trans* isomer with unfolded oligonucleotides. This behavior suggests that binding to flexible unfolded DNA enables ligand molecules to align along the strand in a cooperative and spatially ordered manner, leading to exciton coupling between closely spaced chromophores. In this scenario, the unfolded scaffold acts as a template that organizes the ligands into a defined chiral array rather than a randomly distributed, dynamically averaging ensemble. In contrast, the appearance of an exciton-coupled bisignate ICD signal for complexes formed between antiparallel G4 structures and the *cis*-rich PSS indicates an external binding mode, most likely involving electrostatic interactions along the grooves and/or loop regions, which impose a fixed supramolecular arrangement of the chromophores. By comparison, the absence of a detectable ICD band for parallel G4 structures suggests a different binding geometry, plausibly dominated by end-stacking interactions on the terminal G-quartets, as previously reported for similar systems.^23^

To further probe this mechanism, we performed photoswitching experiments (Figure 3E). The CD spectrum of G4 TBA displayed the characteristic signature of an antiparallel G4 topology, with a positive maximum at 293 nm and a negative band at 270 nm. Upon addition of the *cis*-rich PSS of **exAzoPy**_**2**_, a well-structured exciton-coupled bisignate ICD band emerged. Irradiation at 435 nm induced *cis*-to-*trans* isomerization, resulting in the disappearance of the ICD band. This change is consistent with a shift in the binding mode, likely reflecting the disruption of the previously ordered ligand arrangement. Subsequent irradiation at 550 nm to promote *trans*-to-*cis* isomerization only partially restored the original ICD pattern, indicating limited reversibility under these conditions (Figure 3E). Taken together, these findings reveal a topology- and isomer-dependent chiroptical output arising from distinct binding modes of **exAzoPy**_**2**_ toward different DNA conformations. To the best of our knowledge, this represents the first example of a photoswitchable ligand that translates differences in DNA topology into a measurable ICD response, thereby providing mechanistic insight and a direct spectroscopic readout of structural discrimination.

Finally, to gain further insight into the structural basis of the interaction between **exAzoPy**_**2**_ and antiparallel G4 ^1^H NMR titration experiments were performed using the well-characterized TBA as a model antiparallel G4 (Figure 4). In its unbound state, TBA exhibited eight imino proton resonances, whose assignments have been reported previously.^43^ Upon addition of the *trans* isomer of **exAzoPy**_**2**_, pronounced attenuation of the G1/G10 and G11/G14 imino proton resonances was observed (Figure 4A), indicating that the *trans* isomer predominantly interacts through an end-stacking binding mode (Figure 4C). This interpretation is further supported by the absence of ICD band^44^ and by the relatively weak thermal stabilization observed in the melting experiments.

**Figure 4:**
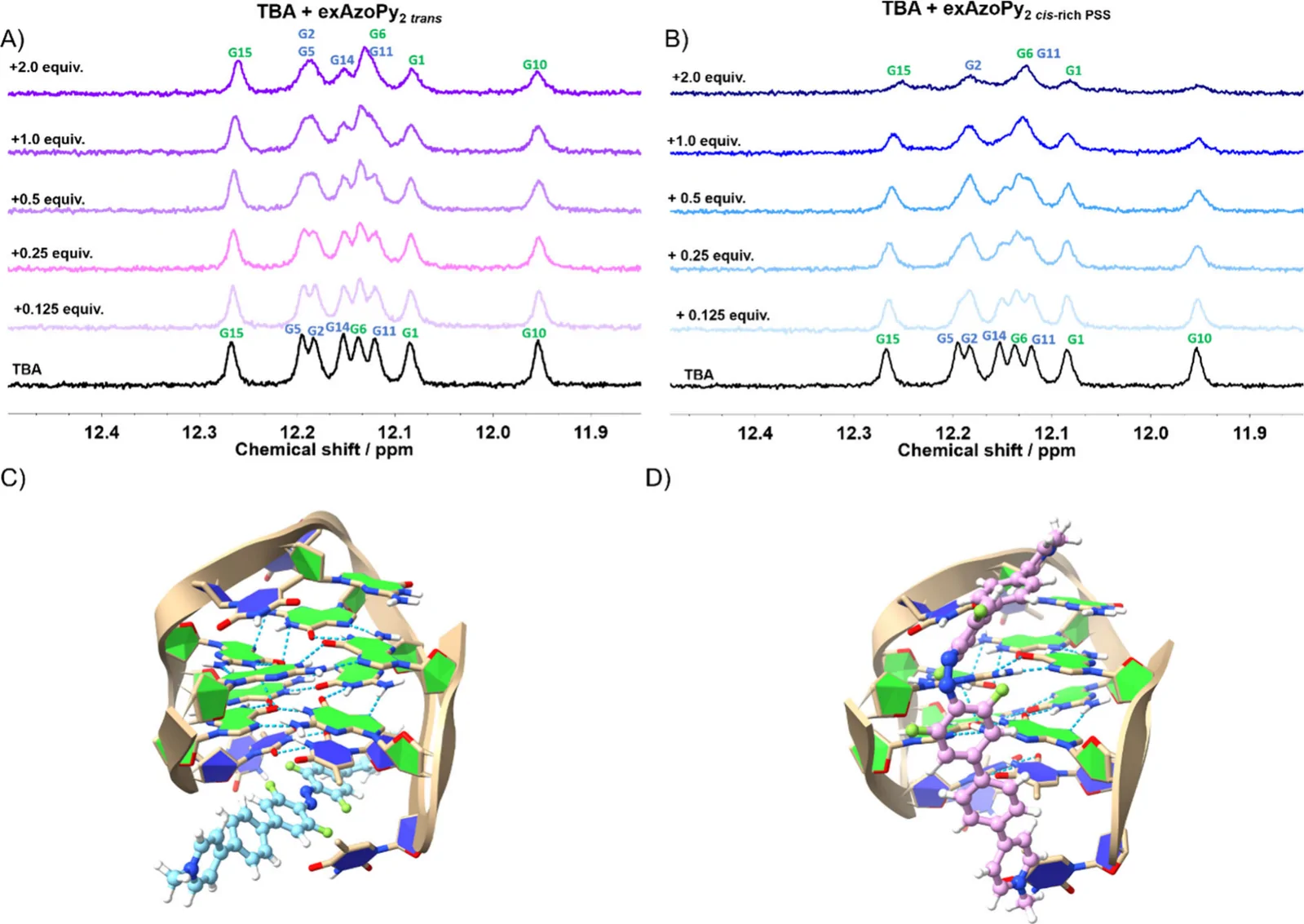
^1^H NMR spectra of the G-tetrad imino protons of G4 TBA in the absence (0.0 equiv) and presence (0.125, 0.25, 0.5, 1.0, and 2.0 equiv) of **exAzoPy**_**2**_ *_trans_* (A) **exAzoPy**_**2**_ *_cis_*_–rich PSS_ (B). Schematic representation of the proposed binding models of TBA (PDB ID: 148D) with *trans*-**exAzoPy**_**2**_ (C) and *cis*-**exAzoPy**_**2**_ (D).

In contrast, titration with the *cis*-rich PSS of **exAzoPy**_**2**_ perturbed most of the imino proton resonances, whereas the resonances assigned to G6 and G11 were affected to a much lesser extent (Figure 4B). These observations indicate that the *cis* isomer adopts a binding mode distinct from that of the *trans* isomer. Based on the observed NMR perturbations together with the CD and thermal melting data, we propose a binding model in which one aromatic arm interacts with the external G-tetrad, whereas the second arm is accommodated in the region between the two G-tetrads, thereby acting as a molecular ″glue″(Figure 4 D). Such a binding mode provides a plausible molecular basis for the enhanced stabilization of the antiparallel G4 and is consistent with its ability to promote folding of the unfolded TBA observed in the CD experiments. This interpretation is further supported by the enhanced thermal stabilization and by the presence of the ICD band,^38,42^ which is characteristic of ligands interacting externally or partially externally with G4 DNA.

G4s represent attractive targets for therapeutic intervention due to their pivotal roles in gene transcription and regulation. Their pronounced structural polymorphism further makes them appealing building blocks for DNA-based materials and nanodevices. Despite their potential, only a limited number of small molecules have been shown to reversibly induce folding and unfolding of G4 structures. Moreover, most reported G4 ligands exhibit poor topological discrimination; when selectivity is observed, it commonly favors parallel over antiparallel G4s, often attributed to favorable stacking interactions at the exposed terminal G-tetrads.^45,46^ Antibodies such as BG4 and a variety of fluorescent probes represent powerful and well-established tools for differentiating between DNA conformations (e.g., ssDNA vs G4) as well as among distinct G4 topologies (parallel vs antiparallel). These fluorescence-based systems provide highly sensitive and convenient readouts. At the same time, the development of small, synthetically accessible, visible-light-responsive ligands that enable reversible and topology-dependent structural discrimination represents an attractive complementary strategy, particularly when dynamic and spatiotemporal control over DNA architecture is desired.

In this work, we designed and synthesized a fully visible-light-responsive G4 DNA ligand, **exAzoPy**_**2**_, based on an *ortho*-fluoroAzo core functionalized in the *para* positions with 1-methyl-4-phenylpyridinium groups to promote interactions with oligonucleotides. While the *ortho*-fluoroAzo scaffold enables visible-light operation, the extended π-system of the substituents induces a red shift of the absorption bands and reduces the separation of the n→π* transitions, leading to a *trans*-rich PSS more characteristic of classical Azo than of conventional *ortho*-fluoro derivatives. Functionally, **exAzoPy**_**2**_ displays isomer-dependent control over antiparallel G4 structures: the *cis* isomer promotes G4 folding, whereas *cis*-to-*trans* photoisomerization favors unfolding toward the unfolded state. Importantly, **exAzoPy**_**2**_ also acts as a photoswitchable chiroptical probe. Depending on its isomeric state and on the DNA conformation (unfolded vs G4 folded) as well as G4 topology (parallel vs antiparallel), distinct ICD responses are generated or suppressed. This topology- and isomer-dependent ICD output provides a direct spectroscopic readout of structural discrimination. Collectively, our results introduce **exAzoPy**_**2**_ as a visible-light-responsive effector and chiroptical reporter capable of modulating and sensing DNA structure in a light-dependent manner. To the best of our knowledge, this represents one of the first examples of a small-molecule photoswitch that combines reversible structural control with topology-sensitive ICD signaling, thereby opening new opportunities for spatiotemporal regulation and characterization of G4 DNA architectures.

## Supplementary Material



## Notes

Experimental procedures, synthesis and characterization of the compounds, photoisomerization and thermal stability studies, G4 DNA-binding studies (PDF)
