# Supplementary material for: Light-Controlled Folding and Chiroptical Sensing of G‑Quadruplex DNA
Source: J Phys Chem Lett. 2026 Jul 31;17(32):9267–75. doi: 10.1021/acs.jpclett.6c01816 (PMC13488439; doi:10.1021/acs.jpclett.6c01816)
Supplement: Supplementary file 1 [file jz6c01816_si_001.pdf]

Supporting Information

Light-Controlled Folding and Chiroptical Sensing of G-Quadruplex DNA

Aleksandra Matusiak, Jakub Trojnar, Katarzyna Matczyszyn, Marco Deiana and Marta Dudek*

Institute of Advanced Materials, Faculty of Chemistry, Wrocław University of Science and Technology Wyb. Wyspiańskiego 27, 50-370 Wrocław, Poland.

Corresponding Author

*E-mail: marta.ziemianek-dudek@pwr.edu.pl

Table of Contents:

1. Materials and methods S3

2. Synthesis and characterization S5

3. Photoisomerization studies S9

4. Thermal stability S9

5. G4 studies S10

    5.1 DNA characterization S10

    5.2 CD spectra of interactions of unfolded DNA with *trans*/*cis*-rich PSS of **exAzoPy$_2$** S11

    5.3 Melting studies of unfolded DNA S12

    5.4 Binding studies S12

    5.5 Melting studies of G4 DNA S13

    5.6 CD spectra of interactions of G4 DNA with *trans*/*cis*-rich PSS of **exAzoPy$_2$** S13

6. References S14

1. MATERIALS AND METHODS

Solvents and reagents

Solvents and reagents were purchased from commercial suppliers and were used without additional purification. Column chromatography was carried out using a flash chromatograph (Teledyne ISCO) equipped with RediSep columns filled with silica gel (particle size: 40–63 μm irregular, mesh size: 230–400) and C18 reverse-phase flash column. Thin Layer Chromatography (TLC) was performed on aluminum sheets coated with silica gel 60 F254 purchased from Merck, and the visualization was done using UV light. Oligonucleotides were purchased from Merck, diluted with Milli-Q water to a concentration of 2 mM, and stored in the fridge. A stock solutions of 1 M KCl in water were prepared using solid salts. The measurements were performed in either 10 mM Tris buffer or 10 mM lithium cacodylate buffer (pH 7.2).

NMR measurements

NMR spectra were recorded on a Bruker Avance^{TM} 600 MHz spectrometer or on a JEOL 400 MHz spectrometer at 25 °C using residual protonated solvent signals as internal standards for ^{1}H, and ^{13}C spectra. Multiplicities are denoted as follows: singlet (s), doublet (d), doublet of doublets (dd), triplet (t), multiplet (m), and broad (br).

High resolution mass spectra

HRMS were conducted with a Waters XEVO-G3QTOF mass spectrometer (ESI) connected with UPLC. Waters ACQUITY QSM

Sample Irradiation

Photoinduced isomerization of **exAzoPy$_2$** was carried out using a UV spot light source (Hamamatsu Photonics K.K., model L9588-04) equipped with optical filters providing irradiation at 366, 405, 435, and ≥ 550 nm.

Absorption spectra

UV-vis absorption experiments were carried out on a JASCO V-730 spectrophotometer equipped with the JascoPeltier type temperature controller (CDF-426S/15) at 25 °C. All optical measurements were performed in quartz cuvettes with path lengths of 10 mm. Changes in the absorption spectra of the 20 μM solutions in 10 mM lithium cacodylate buffer were recorded before and after irradiation with appropriate wavelengths.

Thermal Stability

The thermal relaxation of the *cis* isomer was investigated by monitoring time-dependent absorbance changes at elevated temperatures. A solution of **exAzoPy$_2$** (20 μM) in 10 mM lithium cacodylate buffer (pH 7.2) was first irradiated with light ≥ 550 nm for 10 min to generate the *cis*-rich PSS. Subsequently, UV-vis spectra (220 – 600 nm) were recorded over time at 65, 70, 75, and 80 °C. For each temperature, the rate constant (k) was determined from the time-dependent absorbance changes. An Arrhenius plot of lnk versus 1/T was then constructed, and the thermal half-life of the *cis* isomer at room temperature was calculated using the Arrhenius equation: $\ln k = \ln A - E_a/RT$.

Composition of the photostationary state (PSS)

Photoinduced isomerization of **exAzoPy2** was carried out using a UV Spot Light Source (Hamamatsu Photonics K.K., model L9588-04) equipped with optical filters providing irradiation at 435 and ≥ 550 nm. The compositions of the PSS were determined by ^{1}H NMR spectroscopy. A solution of **exAzoPy2** (3.2 mM in DMSO-d_6 at 25 °C) was first irradiated with light ≥ 550 nm for 15 min to generate the corresponding PSS, after which the ^{1}H NMR spectrum was recorded. The same sample was subsequently irradiated with 435 nm light for 15 min, followed by acquisition of a second ^{1}H NMR spectrum. The *cis*/*trans* ratios at the respective PSS were determined by integration of diagnostic proton signals, and the composition was calculated from the corresponding integral intensity ratios.

Melting studies

DNA melting experiments were performed using a Jasco J-1500 spectropolarimeter (Jasco Inc., USA) equipped with a Peltier-type temperature controller (CDF-426S/15), by monitoring both UV-vis and CD signals. CD melting assays were carried out at a fixed DNA concentration of 2 μM, either in the absence or presence of *trans*- or *cis*-enriched PSS of **exAzoPy2**. The photoswitch concentration was 10 μM for experiments with unfolded oligonucleotides and 6 μM for G4 DNA. Measurements were conducted in 10 mM lithium cacodylate buffer; for G4 samples, the buffer was supplemented with 5 mM KCl. Melting curves were obtained by recording the temperature-dependent changes in CD signal, and melting temperatures (T_m) were determined by sigmoidal nonlinear fitting of the resulting data.

Circular dichroism titration

CD spectra were recorded on a Jasco J-1500 spectropolarimeter (Jasco Inc., USA) equipped with a Peltier-type temperature controller (CDF-426S/15). Measurements were performed at 20 °C in a quartz cuvette with a 10 mm path length, using a scanning speed of 100 nm/min, a data pitch of 1 nm, and a bandwidth of 2 nm. Spectra were collected in the range of 220–600 nm. Prior to measurements, the optical chamber of the spectropolarimeter was purged with nitrogen and maintained under a nitrogen atmosphere throughout the experiments. Appropriate baseline spectra were recorded and subtracted from all CD measurements. CD titration experiments were carried out at a fixed unfolded oligonucleotide concentration (2 μM). Incremental additions of the *cis*-rich PSS of **exAzoPy2** or the *trans* isomer (up to 5 equiv.) were carried out in 10 mM Tris buffer (pH 7.2), unless otherwise stated in the corresponding figure caption. CD experiments on pre-folded G4 structures (2 μM) were performed in the absence or presence of the *trans* isomer or the *cis*-rich PSS of **exAzoPy2** (10 μM) in 10 mM lithium cacodylate buffer (pH 7.2) supplemented with 100 mM KCl.

Fluorescence titration

The apparent dissociation constant (K_d) for *trans* and c*is*-rich mixtures of **exAzoPy2** was determined through fluorescence titration experiments using a JASCO FP-8550 fluorimeter equipped with a Julabo CD-B5 cooling system. The stock solutions of labeled TBA and *c-MYC* Pu22 (Table S2) with Cy5 (Cyanine 5) at 5'-end were prepared in MilliQ water at a 100 μM concentration. The G4 was prefolded in a 10 mM lithium cacodylate buffer (pH 7.2) with 100 mM KCl by heating for 5 minutes at 95 °C and then cooling overnight. The labeled G4 (c_{DNA}= 50 nM, $c_{cacodylate}$ = 10 mM, pH = 7.2, c_{KCl} = 100 mM) was titrated with **exAzoPy2** *trans* and *cis*-rich mixture varied from 1 nM to 20 μM. The complex G4-ligand was allowed to

equilibrate for 3 min before recording the emission spectra (λ_{ex} = 645 nm; λ_{em} = 650-800 nm). Binding constants were calculated by fitting the data to the Dose-Resp equation in OriginPro 2020b.

^{1}H NMR titration

The experiments were carried out with TBA (70 mM KCl) in lithium cacodylate buffer (10 mM, 10% D_2O, pH = 7.2) with the final concentration of 200 µM. G4s were titrated with increasing amounts of *trans* and *cis*-rich mixture from a 20 mM DMSO-*d*6 stock solution. Spectra were recorded at 298 K on a Bruker 600 MHz spectrometer. Excitation sculpting was used in the ^{1}H NMR experiments, and 256 scans were recorded.

2. SYNTHESIS AND CHARACTERIZATION

Synthesis of **exAzoPy₂**

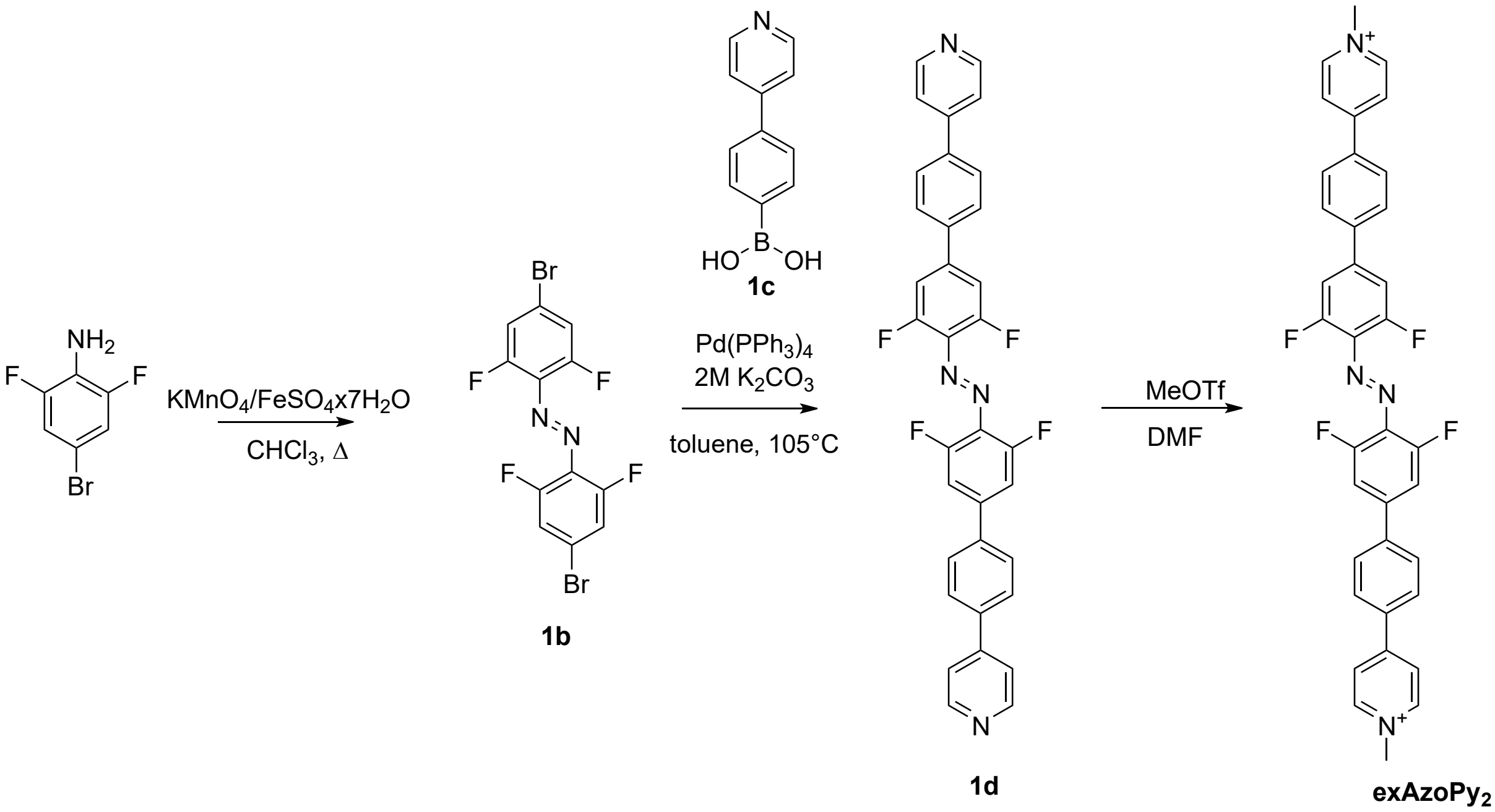

Scheme S1. Synthetic route of photoswitch **exAzoPy$_2$**.

1b To the solution of 4-bromo-2,6 difluoroaniline (5.0 g, 24 mmol, 1.0 equiv.) in chloroform (60 mL), the mixture of $KMnO_4$ (10 g, 63 mmol, 2.6 equiv.) and $FeSO_4 \cdot 7H_2O$ (10 g, 36 mmol, 1.5 equiv.) was added. The solution was refluxed overnight, filtered through Celite®, dried over $MgSO_4$, and concentrated under reduced pressure. The crude residue was purified by column chromatography (DCM/hexane mixtures). The product was obtained as a red solid (1.1 g, 22%).

1b - The spectroscopic data are in accordance with the literature.[1]

1d *Suzuki coupling*[2]

To a stirred solution of **1b** (0.2 g, 0.49 mmol, 1 equiv.) and (4-(pyridin-4-yl)phenyl)boronic acid (0.29 g, 1,47 mmol, 3 equiv.) in toluene (15 mL), an aqueous solution of base (0.735 mL, 3 equiv.) was added, and the resulting suspension was degassed with argon for 15 min. The palladium catalyst (0.057 g, 0.049 mmol, 0.1 equiv.) was then introduced, and the mixture was

further degassed for 5 min before being heated at 105 °C overnight under an argon atmosphere. After completion of the reaction, the mixture was filtered through a pad of Celite®, which was subsequently washed with DCM (30 mL). The combined filtrates were washed with water (3 × 50 mL) and brine (50 mL). The organic phase was dried over anhydrous Na_2SO_4, and the solvent was removed under reduced pressure. The crude residue was carried forward to the next step without further purification. The product was obtained as a red solid.

HRMS m/z (ESI): $C_{34}H_{20}F_4N_4$: $[M+H]^+$, calculated: 561.1697, found: 561.1693.

exAzoPy₂ *Methylation*

To a solution of **1d** (1 equiv.) in DMF, the methylating agent, MeOTf (3 equiv.) was added dropwise. The reaction mixture was stirred at room temperature overnight. The crude product was purified by flash column chromatography on a C18 column using a gradient elution from water to water/MeCN (40:60, + 0.1% TFA) over 15 min, affording the corresponding TFA salt. Subsequently, toluene (10 mL) and methanol (20 mL) were added, and the solvents were evaporated again to facilitate complete removal of residual TFA. During evaporation, the product precipitated as methanol was removed, while toluene remained in the round-bottom flask. The resulting solid was collected by filtration, washed thoroughly with diethyl ether, and dried under reduced pressure. **exAzoPy$_2$** was obtained as a red solid in 25% yield over two steps.

HRMS m/z (ESI): calc. for: $C_{36}H_{26}F_4N_4^{2+}$: $[M/2]^+$, required: 295.1047, found: 295.1049.

^{1}H NMR (400 MHz, DMSO-d_6) δ: 9.05 (d, *J* = 6.7 Hz, 4H), 8.63 (d, *J* = 7.0 Hz, 4H), 8.27 (d, *J* = 8.7 Hz, 4H), 8.20 (d, *J* = 8.6 Hz, 4H), 7.99 (d, *J* = 10.8 Hz, 4H), 4.35 (s, 6H).

^{19}F NMR (376 MHz, DMSO-d_6) δ: -73.32 (TFA, 6F), -119.96 (d, *J* = 11.6 Hz, 4F).

^{13}C NMR (151 MHz, DMSO-d_6) δ: 155.3 (dd, *J* = 259.2, 5.1 Hz), 153.2, 145.7, 143.1 (t, *J* = 10.5 Hz), 139.6, 134.2, 130.1 (t, *J* = 9.9 Hz), 128.8, 128.2, 124.2, 111.5 (dd, *J* = 21.3, 3.2 Hz), 47.1.

Melting point: Decomp. > 197 °C.

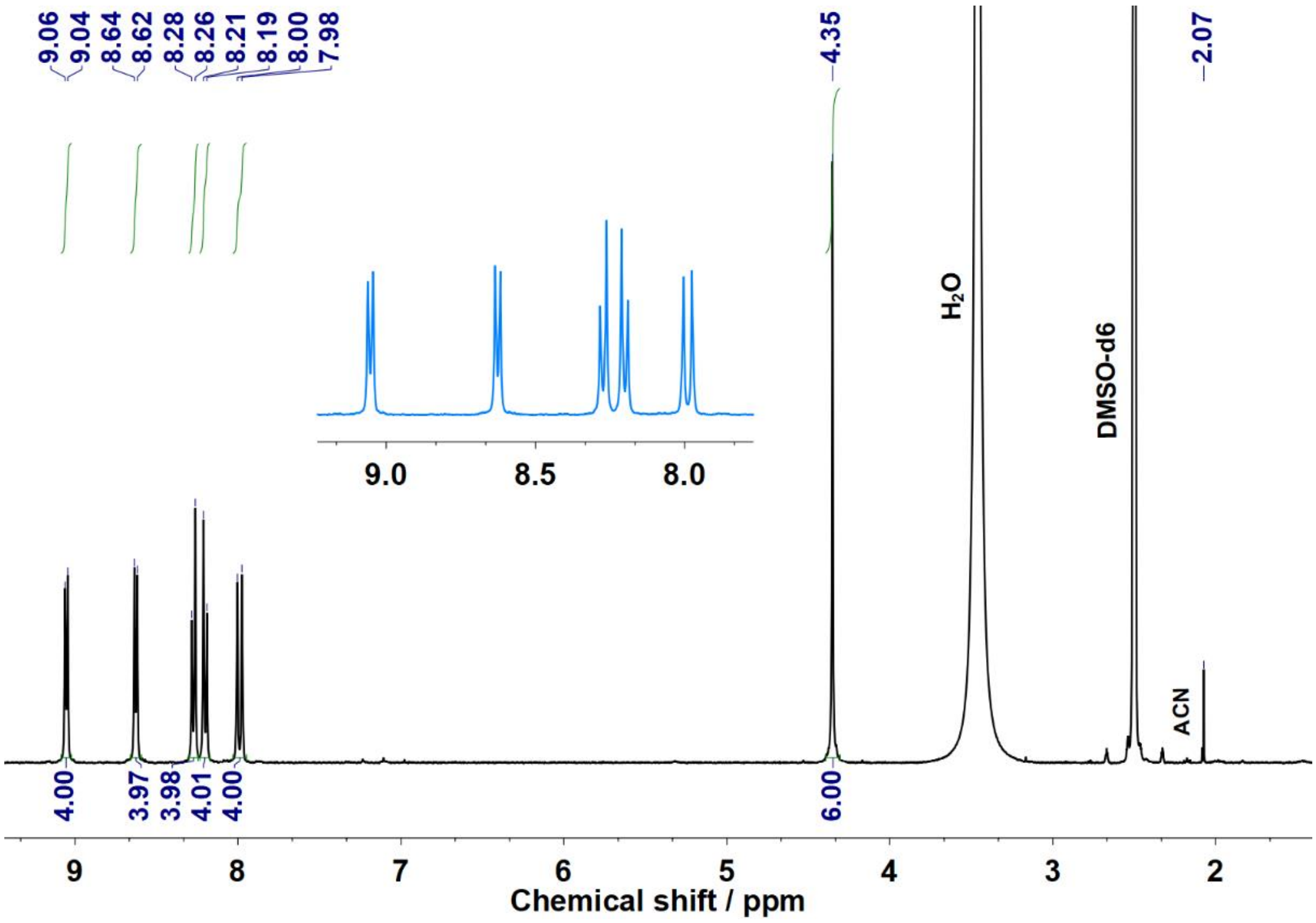

Figure S1. ^{1}H NMR spectrum of **exAzoPy$_2$**.

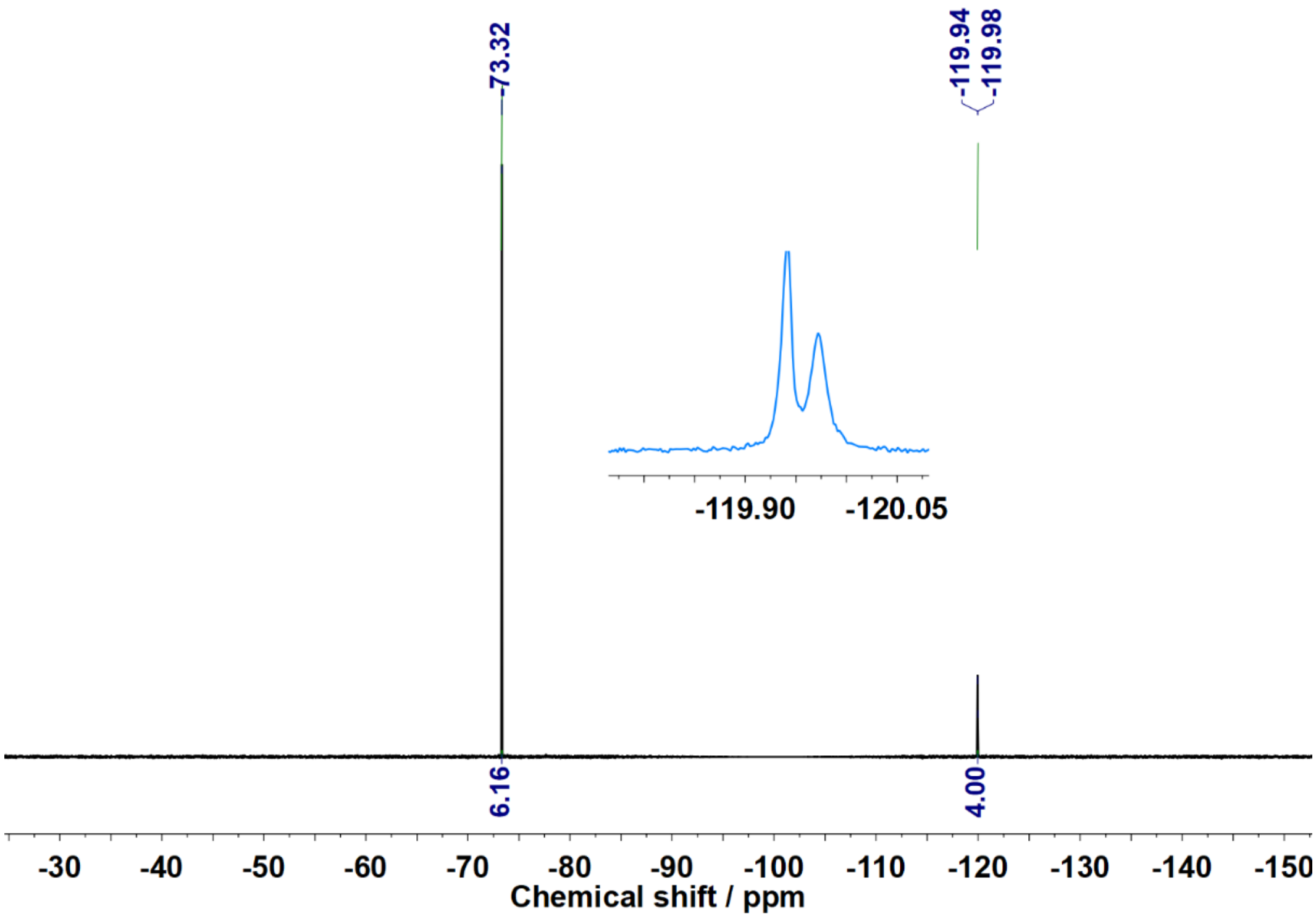

Figure S2. ^{19}F NMR spectrum of **exAzoPy$_2$**.

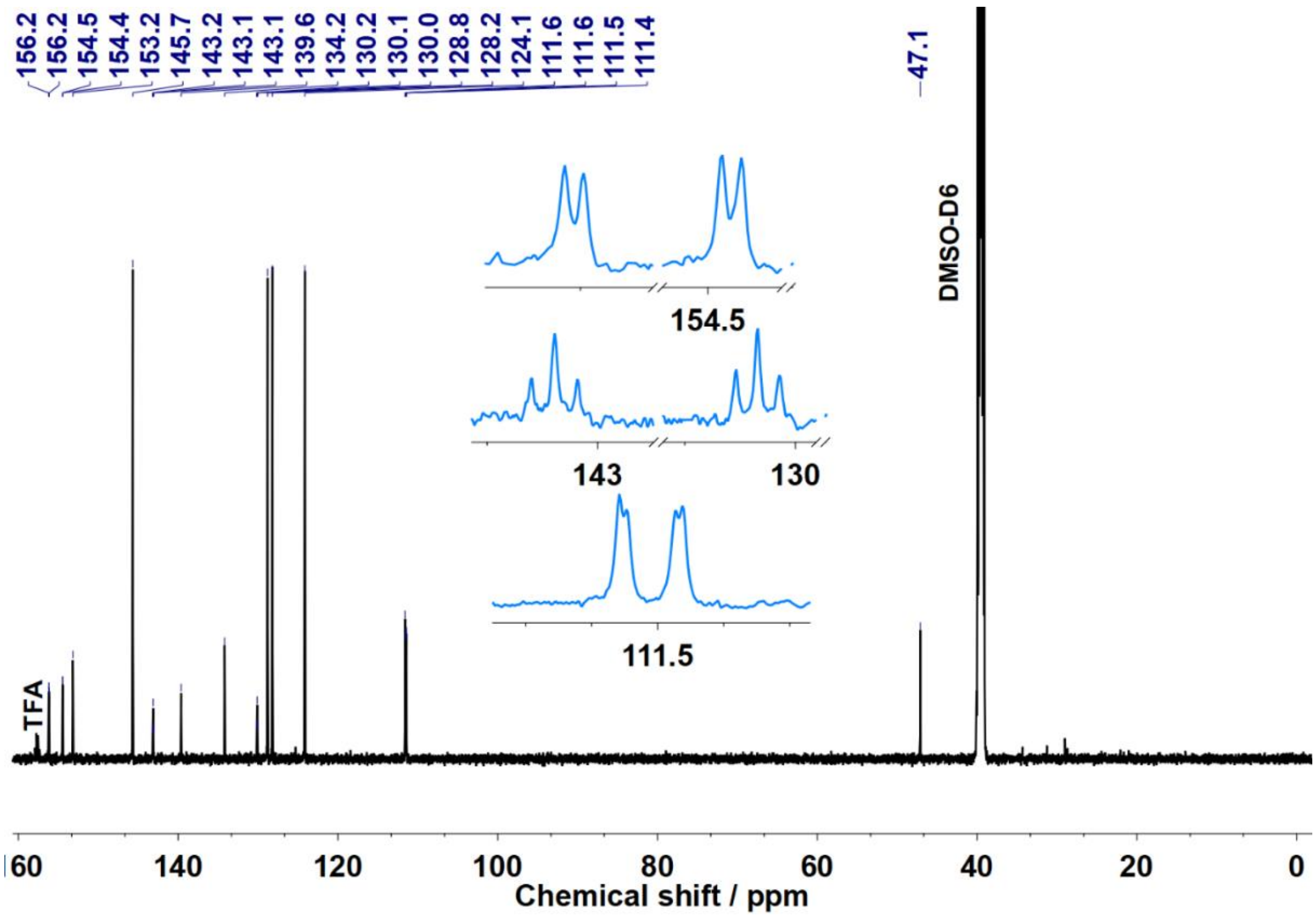

Figure S3. ^{13}C NMR spectrum of **1exAzoPy2**.

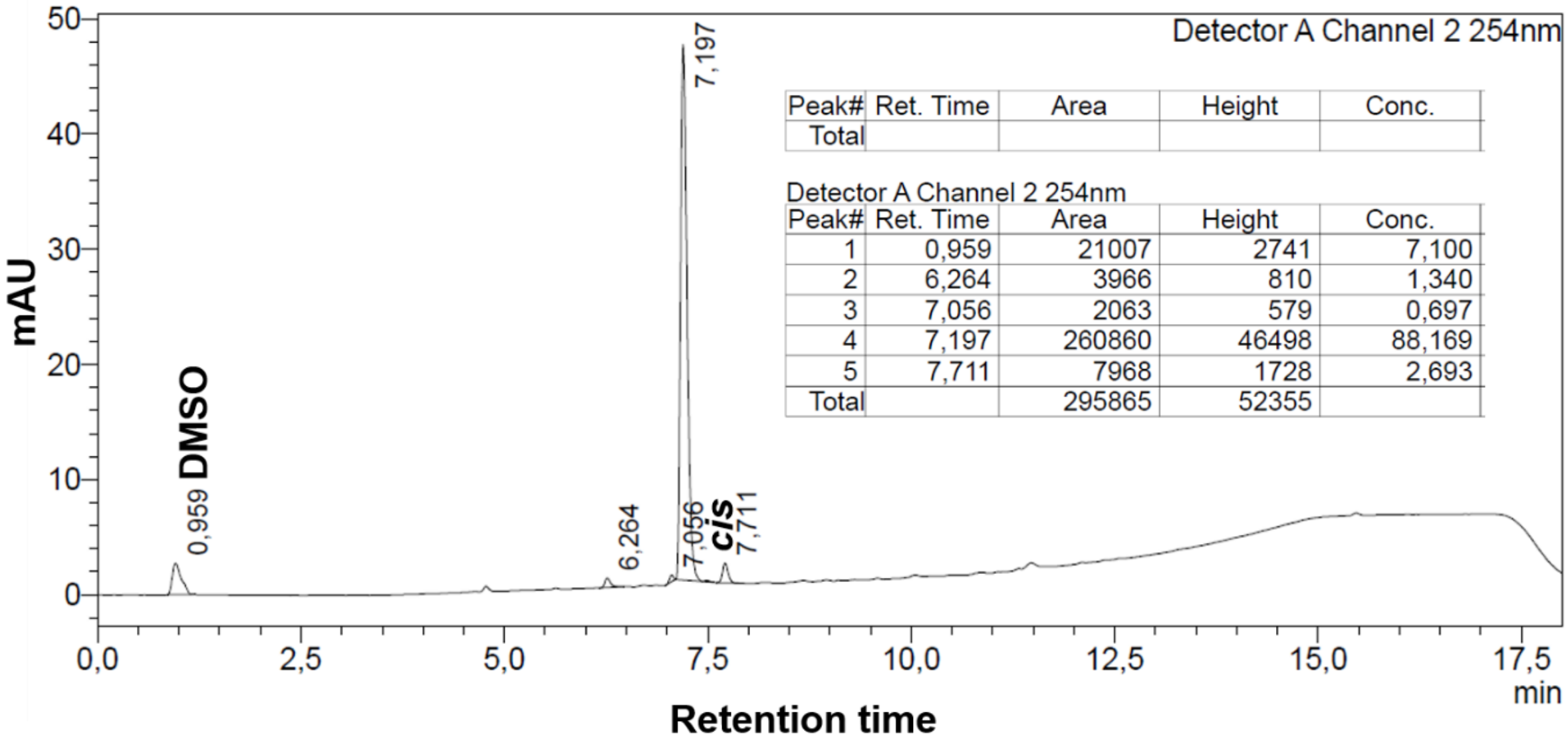

Figure S4. HPLC traces at 254 nm of compound **exAzoPy2** at 298 K. The purity of **exAzoPy2** was determined to be 98%. The signal at 0.959 min corresponds to DMSO, while the peak at 7.711 min is attributed to the *cis* isomer.

3. PHOTOISOMERIZATION STUDIES

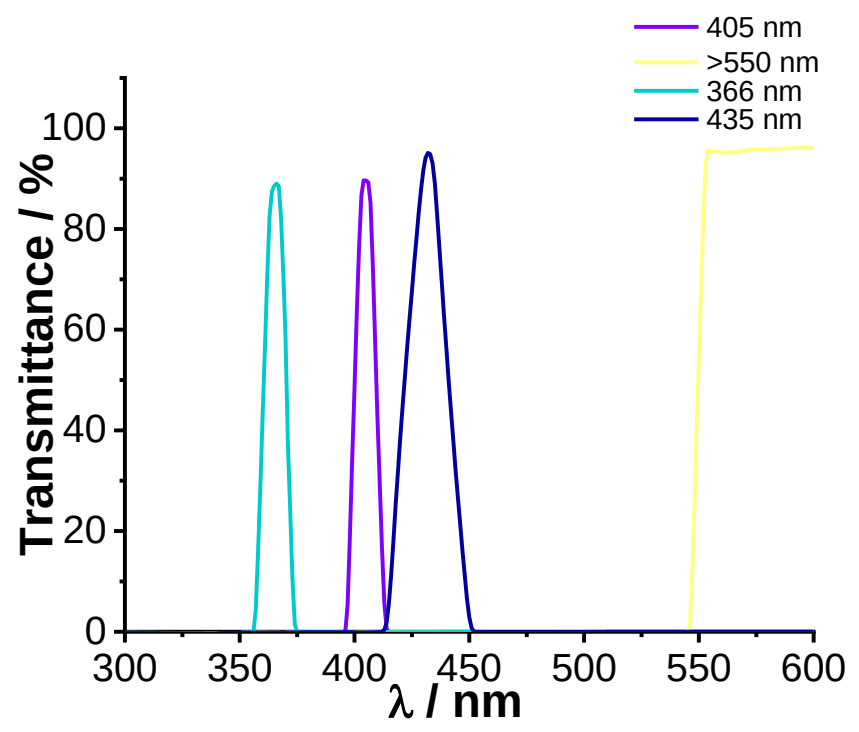

Figure S5. Transmission spectra of the filters used in this study.

4. THERMAL STABILITY

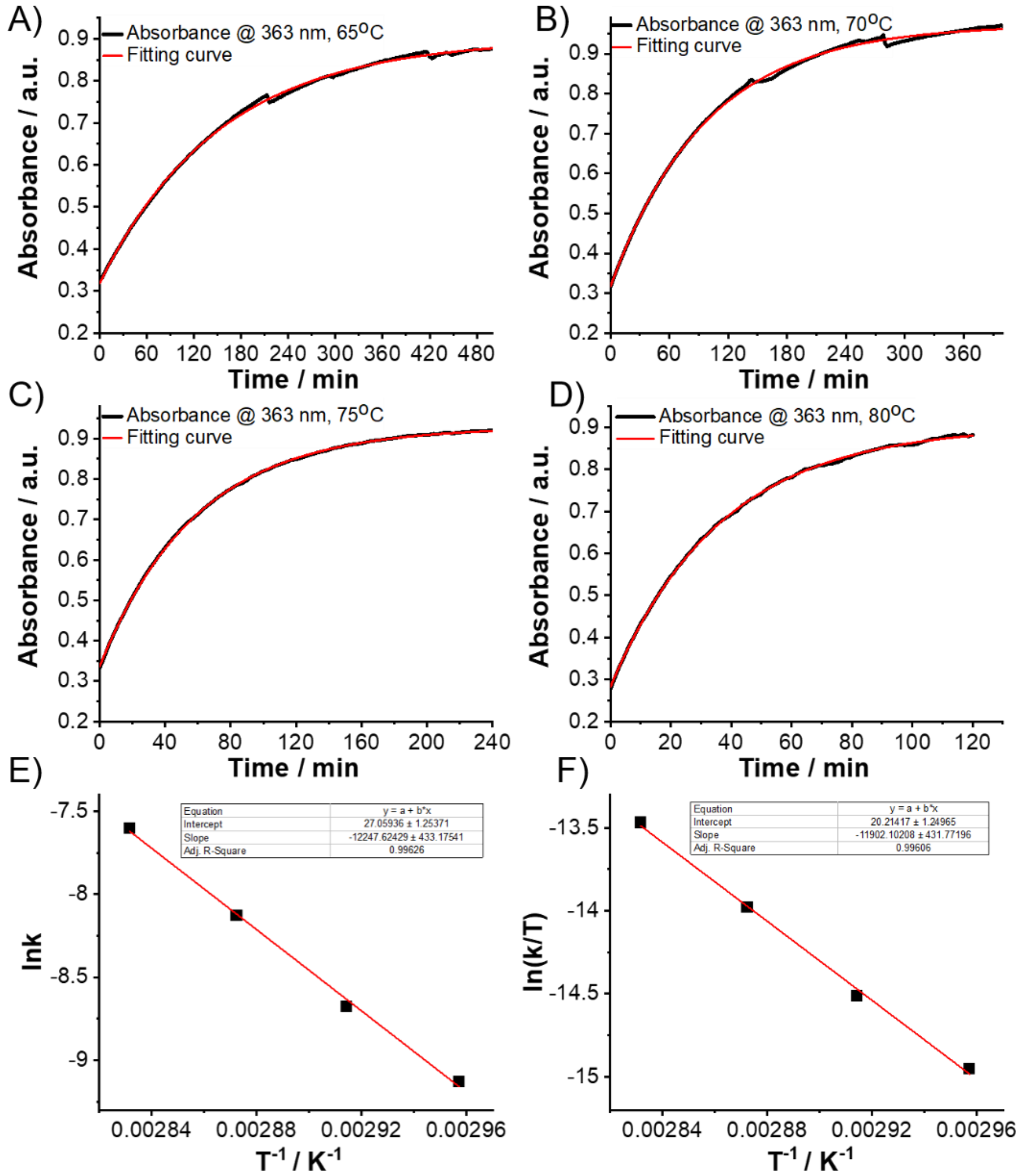

Figure S6. The absorbance changes of 20 μM solution of **exAzoPy2** in 10 mM cacodylate buffer were monitored at 363 nm during *cis–trans* thermal relaxation at four temperatures: 65 (A), 70 (B), 75 (C), and 80 (D) °C. (E) Arrhenius plot for **exAzoPy2** (the rate constants of the

cis-trans isomerization process at different temperatures were determined by the exponential curve fitting of the plot: absorbance vs. time). The slope is equal to -Ea/R. (F) Eyring plot for **exAzoPy**$_2$, slope is equal to -ΔH‡ /R, intercept: ln((κkB)/h) + ΔS‡ /R

Table S1. Isomerization rate $k_{cis \rightarrow trans}$ (at 298 K), thermal half-life ($\tau_{1/2}$) (at 298 K), Arrhenius prefactor A, activation energies E_a, as well as Eyring activation free energies ΔG‡, enthalpies ΔH‡ and entropies ΔS‡ for **exAzoPy**$_2$ in 10 mM lithium cacodylate buffer solution.

**Cmpd**	$k_{E\rightarrow Z}^{[a]}$ [s^{-1}]	$lnk_{E\rightarrow Z}$	$\tau_{1/2}^{[b]}$ [h]	A[c] [s^{-1}]	Ea $[\frac{kJ}{mol}]$	ΔH‡ $[\frac{kJ}{mol}]$	ΔS‡ $[\frac{J}{mol \times K}]$	ΔG‡ $[\frac{kJ}{mol}]$
**exAzoPy**$_2$	8.16	-14.0	236	5.65	101.8	99.0	-29.5	107.8

[a] 10^{-7} at 298 K and [b] at 298 K, estimated from the Arrhenius equation. [c] 10^{11}

5. G4 STUDIES

5.1 DNA CHARACTERIZATION

Synthetic oligonucleotides were purchased from MERCK. The sequences used are listed in Table S2. All the oligonucleotides were prefolded in 100 mM KCl by heating for 5 min at 95 °C and then cooling overnight.

Table S2. Oligonucleotides used in this study.

**Name**	**Sequence [5' – 3']**	**Length (bp)**	**Topology and description**
**Bom17**	GGTTAGGTTAGGTTAGG	17	antiparallel
**TBA**	GGTTGGTGTGGTTGG	15	antiparallel
**Cy5'-TBA**	Cy5[a]-GGTTGGTGTGGTTGG	15	antiparallel
**VEGF**	GGGAGGGTTGGGGTGGG	17	parallel
c-MYC **Pu22**	TGAGGGTGGGTAGGGTGGGTAA	22	parallel
**Cy5'-*c-MYC*** **Pu22**	Cy5[a]-TGAGGGTGGGTAGGGTGGGTAA	22	parallel
c-MYC **Pu24-T**	TGAGGGTGGTGAGGGTGGGGAAGG	24	parallel

[a] Cy5 - Cyanine5

5.2 CD SPECTRA OF INTERACTIONS OF UNFOLDED DNA WITH *TRANS*/*CIS*-RICH PSS OF exAzoPy$_2$

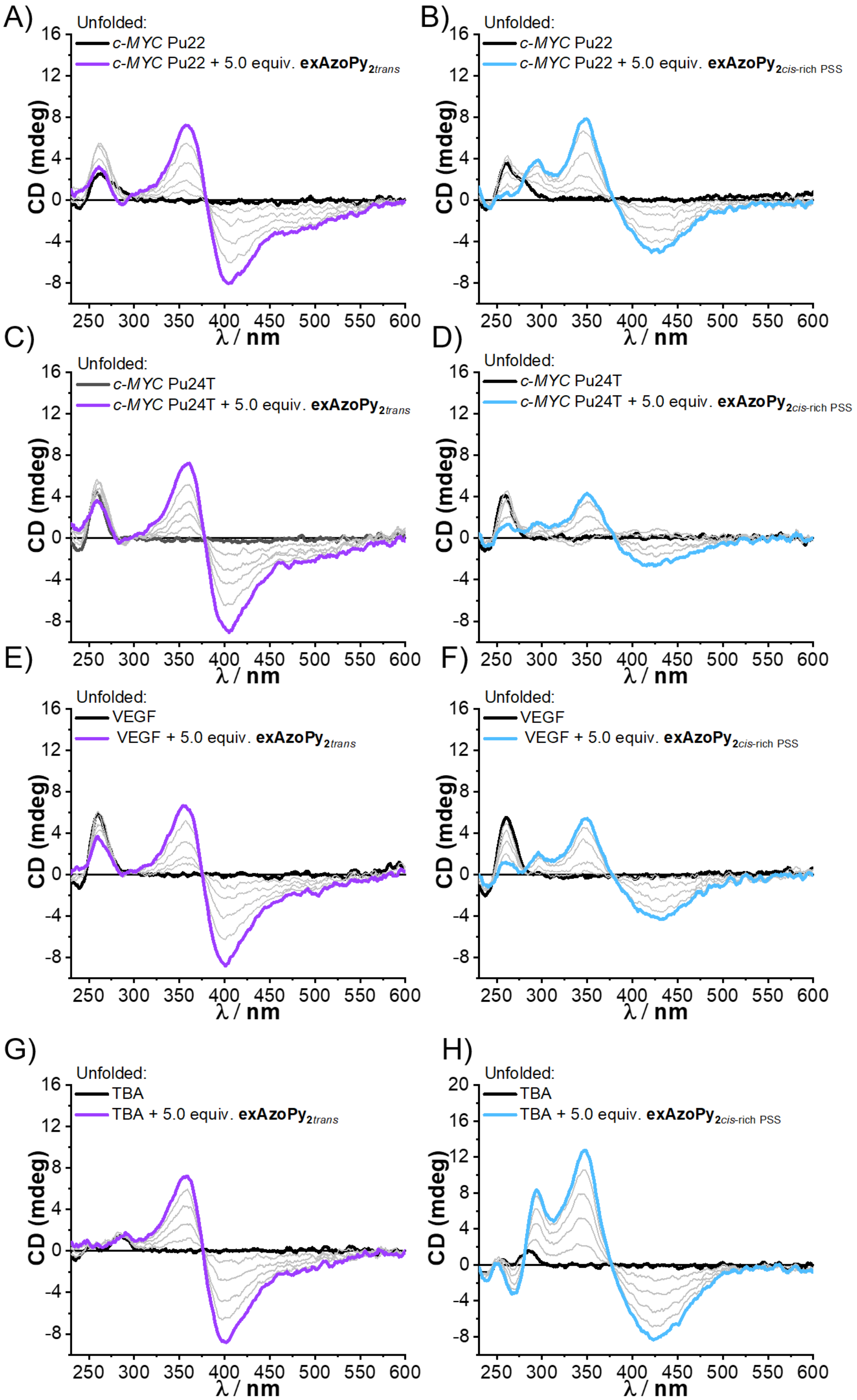

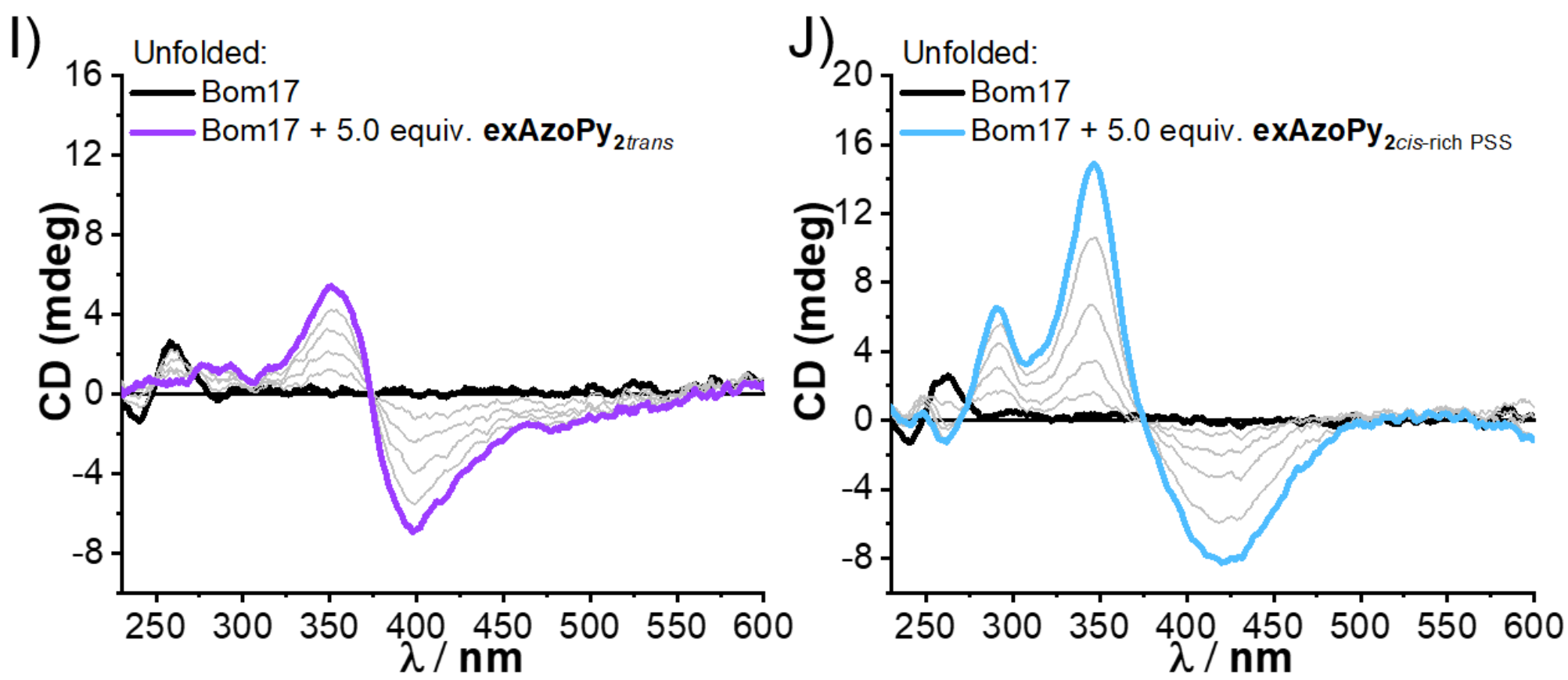

Figure S7. CD spectra of the unfolded *c-MYC* Pu22 (A, B), *c-MYC* Pu24T (C, D), VEGF (E, F), TBA (G, H), and Bom17 (I, J), recorded in the absence and in the presence of the *trans* (A, C, E, G, I)/*cis*-rich PSS (B, D, F, H, J) of **exAzoPy2**. Experimental conditions: c_{DNA} = 2 μM, c_{tris} = 10 mM, $c_{photoswitch}$ = 10 μM.

5.3 MELTING STUDIES OF UNFOLDED AND FOLDED DNA

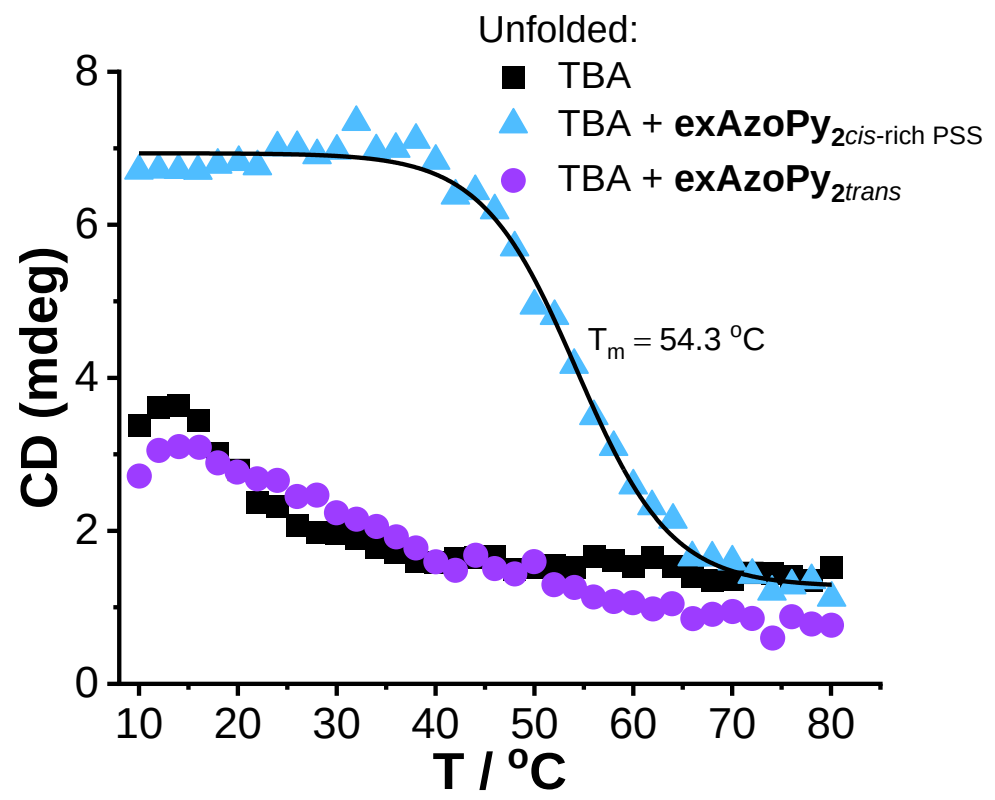

Figure S8. Melting profiles of unfolded TBA alone and in the presence *trans*/*cis*-rich PSS of **exAzoPy2**. Experimental conditions: c_{DNA} = 2 μM, $c_{cacodylate}$ = 10 mM, $c_{photoswitch}$ = 10 μM. The CD signal was plotted as a function of temperature at the maximum wavelength (293 nm).

5.4 BINDING STUDIES

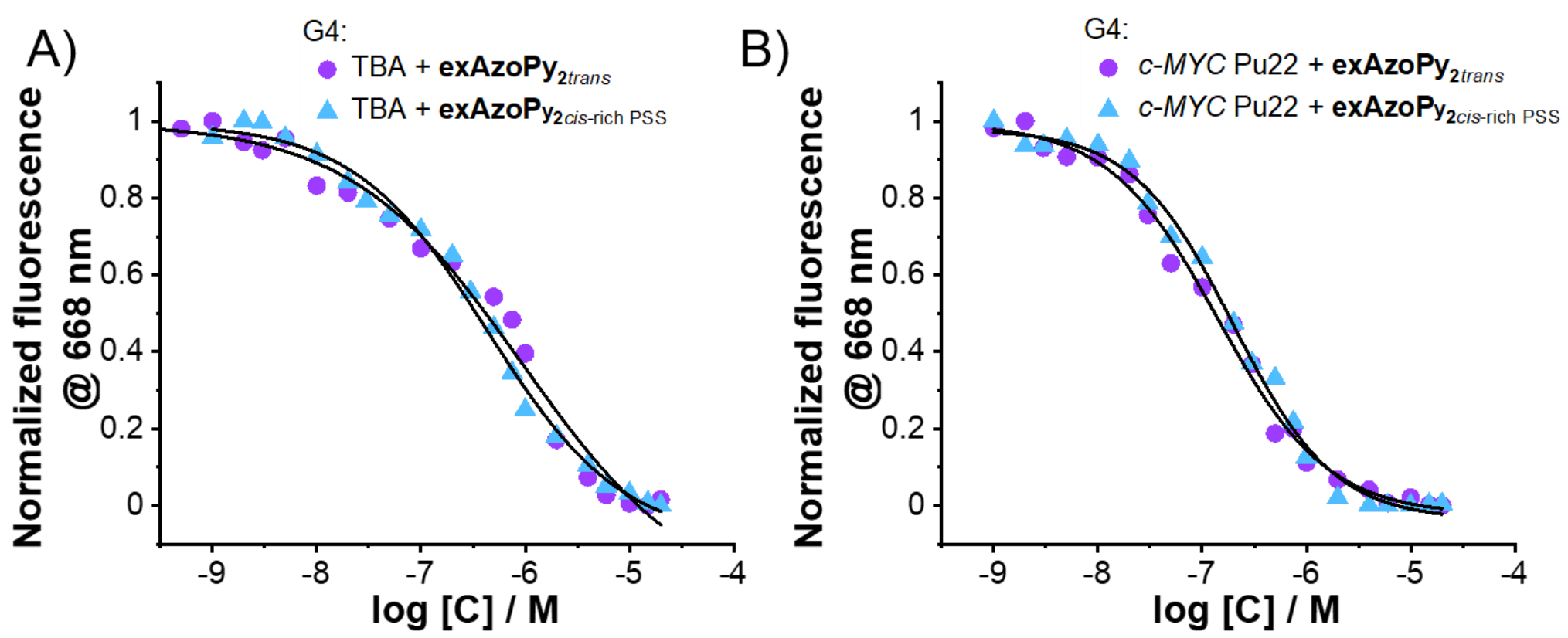

Figure S9. Fluorescence changes of (A) Cy5-TBA G4 or (B) Cy5-*c-MYC* Pu22 (c_{G4} = 50 nM, 10 mM lithium cacodylate buffer pH 7.2, 100 mM KCl, 298 K) in the presence of the

incremental addition of **exAzoPy2** in either *trans* (violet symbols) or *cis*-rich PSS (blue symbols). Black lines represent the best data fits.

5.5 MELTING STUDIES OF G4 DNA

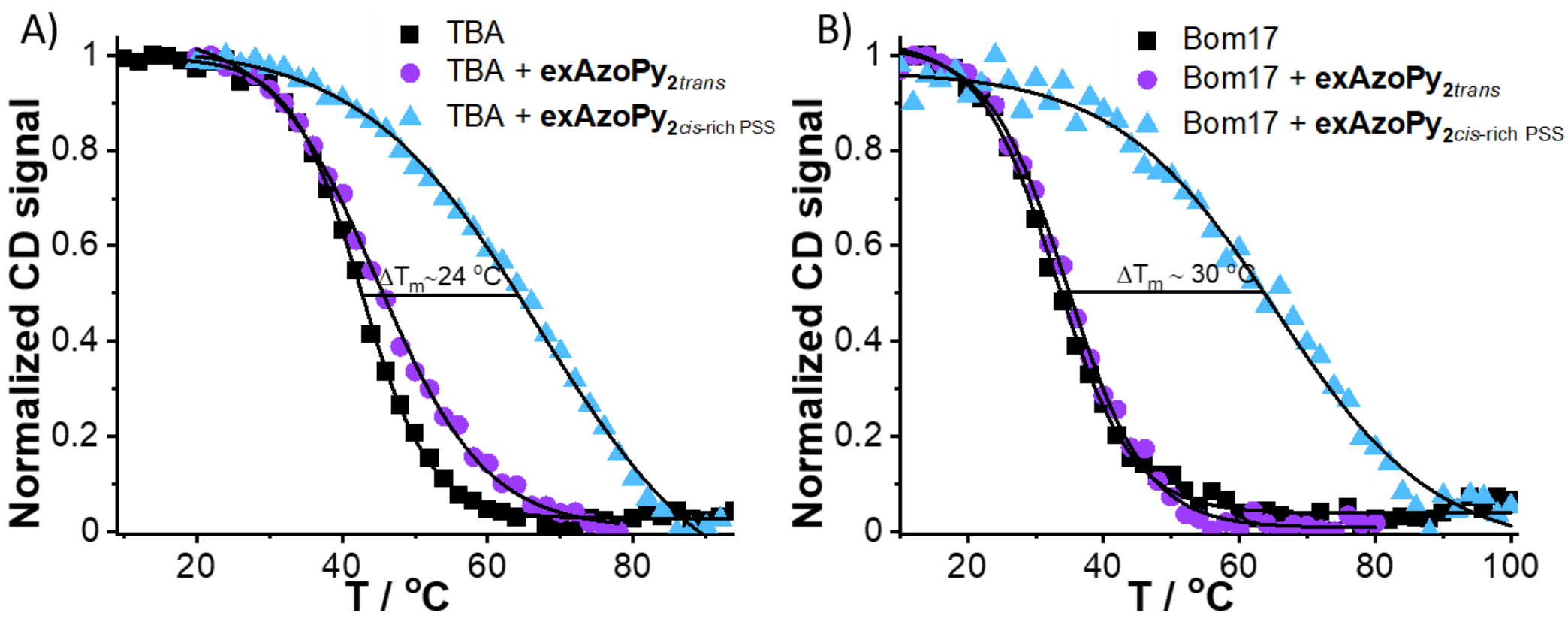

Figure S10. Melting curves for TBA (A), and Bom17 (B), in the absence and presence of *trans*/*cis*-rich PSS of **exAzoPy2** ($c_{photoswitch}$ = 6.0 µM, c_{G4} = 2.0 µM, c_{KCl} = 5 mM, $c_{cacodylate}$ = 10 mM pH 7.2. The CD signal was plotted as a function of temperature at the maximum wavelength for each DNA sequence: TBA: 294 nm, Bom17: 292 nm.

5.6 CD SPECTRA OF INTERACTIONS OF G4 DNA WITH *TRANS*/*CIS*-RICH PSS OF exAzoPy2

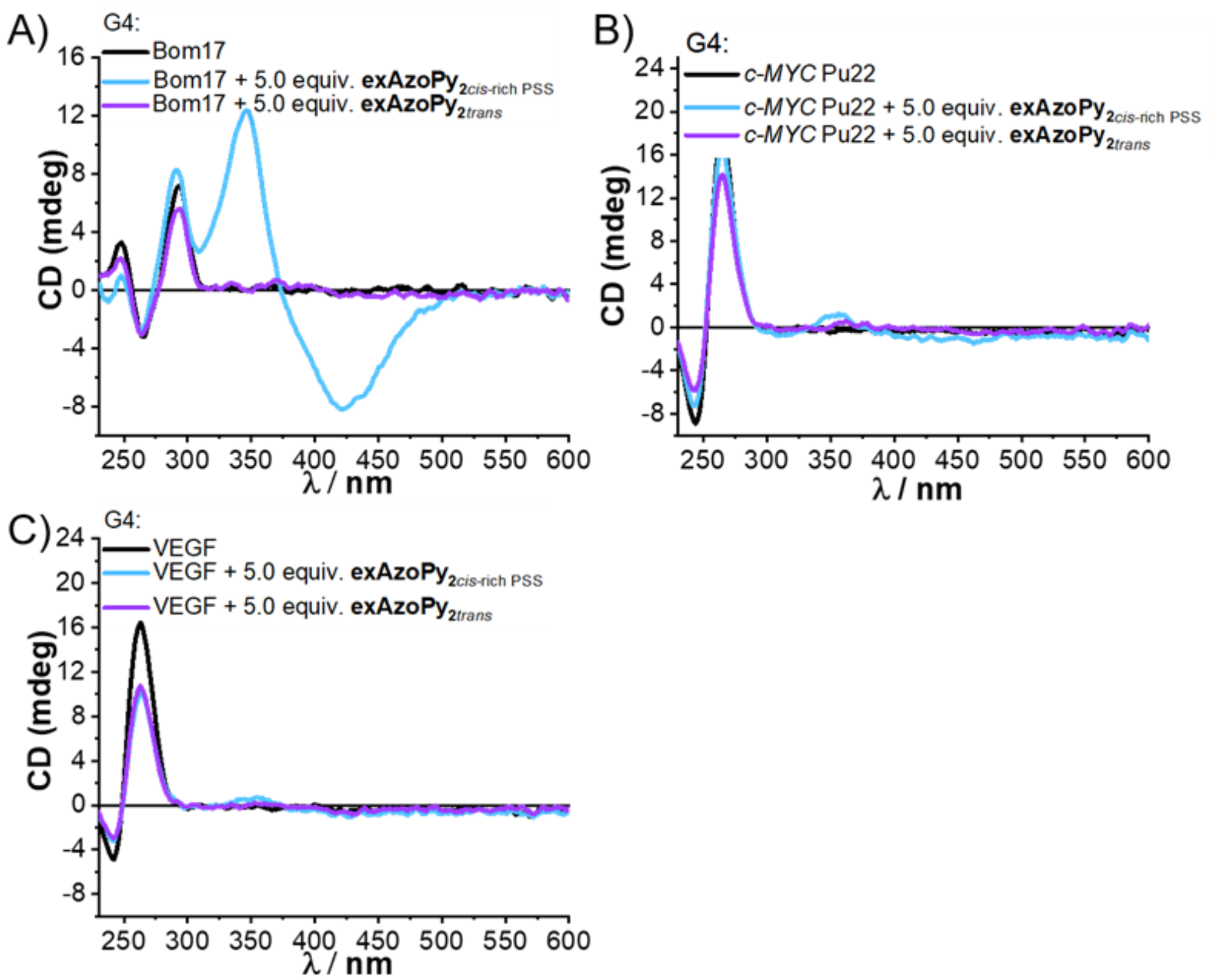

Figure S11. CD spectra of the G4 of Bom17 (A), *c-MYC* Pu22 (B), VEGF (C), recorded

in the absence and in the presence of the *trans*/*cis*-rich PSS of **exAzoPy$_2$**. Experimental conditions: c_{G4} = 2 μM, $c_{cacodylate}$ = 10 mM, c_{KCl} = 100 mM, $c_{photoswitch}$ = 10 μM.

6. REFERENCES

1. Bléger, D.; Schwarz, J.; Brouwer, A. M.; Hecht, S., o-Fluoroazobenzenes as Readily Synthesized Photoswitches Offering Nearly Quantitative Two-Way Isomerization with Visible Light. *J. Am. Chem. Soc.* **2012,** *134* (51), 20597-20600.
2. Matusiak, A.; Drąg, M.; Deiana, M.; Janicki, M. J.; Dudek, M., Tunable G-Quadruplex Ligands: Azobenzene Derivatives for Light-Controlled DNA Modulation. *Chem. Eur. J.* **2025,** *31*, e202404365.
